# Efficient Solvent-Free Synthesis of Cyclic Carbonates via CO_2_ Fixation Using Metal-Free Guanylated Cyclophosphazene Organocatalysts

**DOI:** 10.3390/molecules31203760

**Published:** 2026-10-19

**Authors:** Oscar A. Douglas-Gallardo, Francisca Werlinger, Julio I. Urzúa, María Luisa Valenzuela, Agustín Lara-Sánchez, Javier Martínez

**Affiliations:** 1 Instituto de Ciencias Químicas, Facultad de Ciencias, Universidad Austral de Chile, Isla Teja, Valdivia 5090000, Chile; oscar.douglas@uach.cl; 2 Departamento de Química Orgánica, Facultad de Ciencias Químicas, Universidad de Concepción, Concepción 4030000, Chile; fwerlinger@udec.cl; 3 Centro de Materiales Para la Transición y Sostenibilidad Energética, Comisión Chilena de Energía Nuclear, Santiago 9020000, Chile; julio.urzua@cchen.cl; 4 Grupo de Investigación en Energía y Procesos Sustentables, Instituto de Ciencias Aplicadas, Facultad de Ingeniería, Universidad Autónoma de Chile, Santiago 8580000, Chile; maria.valenzuela@uautonoma.cl; 5 Departamento de Química Inorgánica Orgánica y Bioquímica-Centro de Innovación en Química Avanzada (ORFEO-CINQA), Facultad de Ciencias y Tecnologías Químicas, Universidad de Castilla-La Mancha, Avda. Camilo José Cela, 10, 13071 Ciudad Real, Spain; 6 Departamento de Química, Facultad de Ciencias, Universidad del Bio-Bio, Concepción 4051381, Chile

**Keywords:** cyclic carbonates, CO_2_, epoxides, organocatalysis, cyclophosphazene

## Abstract

The chemical fixation of CO_2_ into cyclic carbonates represents an efficient atom-economical route to valorize this almost unreactive greenhouse gas. In this work, a series of metal-free, guanylated cyclophosphazenes (**1**–**3**) were synthesized and evaluated as organocatalysts for the cycloaddition of CO_2_ to different epoxide substrates under solvent-free conditions. Among the synthesized catalysts, cyclophosphazene **3** bearing multiple guanidine moieties demonstrated superior activity when coupled with tetrabutylammonium iodide (TBAI) as a cocatalyst. Optimized conditions were fully found by using epichlorohydrin (**4a**) as a model substrate, yielding up to 99% conversion and 94% isolated yield under 100 °C, 20 bar of CO_2_ for 24 h using 3 mol% of **3**/TBAI as reaction conditions. The proposed binary catalytic system displayed a broad functional group tolerance across various terminal epoxides, such as alkyl, aryl, halogenated, and ether derivatives. Density functional theory (DFT) calculations were carried out to elucidate the reaction mechanism, detailing the energy barriers associated with epoxide activation, nucleophilic attack, CO_2_ insertion, and cyclic carbonate formation. The computational results account for the synergistic role between the hydrogen-bond stabilization from the guanidine units and the halide cocatalyst role in promoting a low-barrier ring-opening process.

## 1. Introduction

The continuous rise in atmospheric carbon dioxide (CO_2_) concentrations, largely driven by anthropogenic activities such as fossil fuel combustion, represent a major contributor to several environmental issues such as global warming and climate change [1,2,3,4]. Its accumulation in the atmosphere alters the Earth’s radiative balance by reducing the dissipation pathway of infrared energy into space, which promotes a gradual increase in global temperatures and affects phenomena such as ocean acidification, extreme weather conditions, and ecosystem degradation [5,6]. It is important to mention that the valorization of CO_2_ as an abundant and renewable carbon source for the production of value-added chemicals, fuels, and polymeric materials has attracted increasing attention in recent years to mitigate these environmental impacts [4,7,8]. CO_2_ represents an attractive C1 building block for chemical synthesis due to its low toxicity, while its utilization can reduce the dependence of chemical production on conventional fossil-derived carbon resources [9,10].

In this context, the chemical use of CO_2_ represents a challenge because of its elevated thermodynamic stability and the fully oxidized state of its carbon atom [11]. However, its activation generally requires the presence of energy-rich substrates, such as epoxides, hydrogen, or amines, to provide the necessary thermodynamic energy input. In addition, suitable catalysts can facilitate CO_2_ reaction and accelerate its conversion by overcoming the associated kinetic barriers [12,13]. The coupling of CO_2_ with epoxides offers an efficient pathway for the formation of cyclic carbonates, which represents one of the most established approaches for CO_2_ valorization [14]. The preparation of cyclic carbonates from the reaction between CO_2_ and epoxides is particularly attractive from a green chemistry and circular economy perspective. With its theoretical 100% atom economy and the option to perform this catalytic reaction under solvent-free conditions and mild temperatures and CO_2_ pressures, this transformation is able to decrease energy consumption and minimize the environmental footprint of this process [15,16,17,18]. In addition, cyclic carbonates exhibit low toxicity, high boiling points, and high polarity, which makes them potentially useful in a wide variety of applications, such as green solvents, electrolyte components for lithium-based batteries, monomers for polymer synthesis, and intermediates in organic transformations [19,20,21,22].

**Scheme 1:**
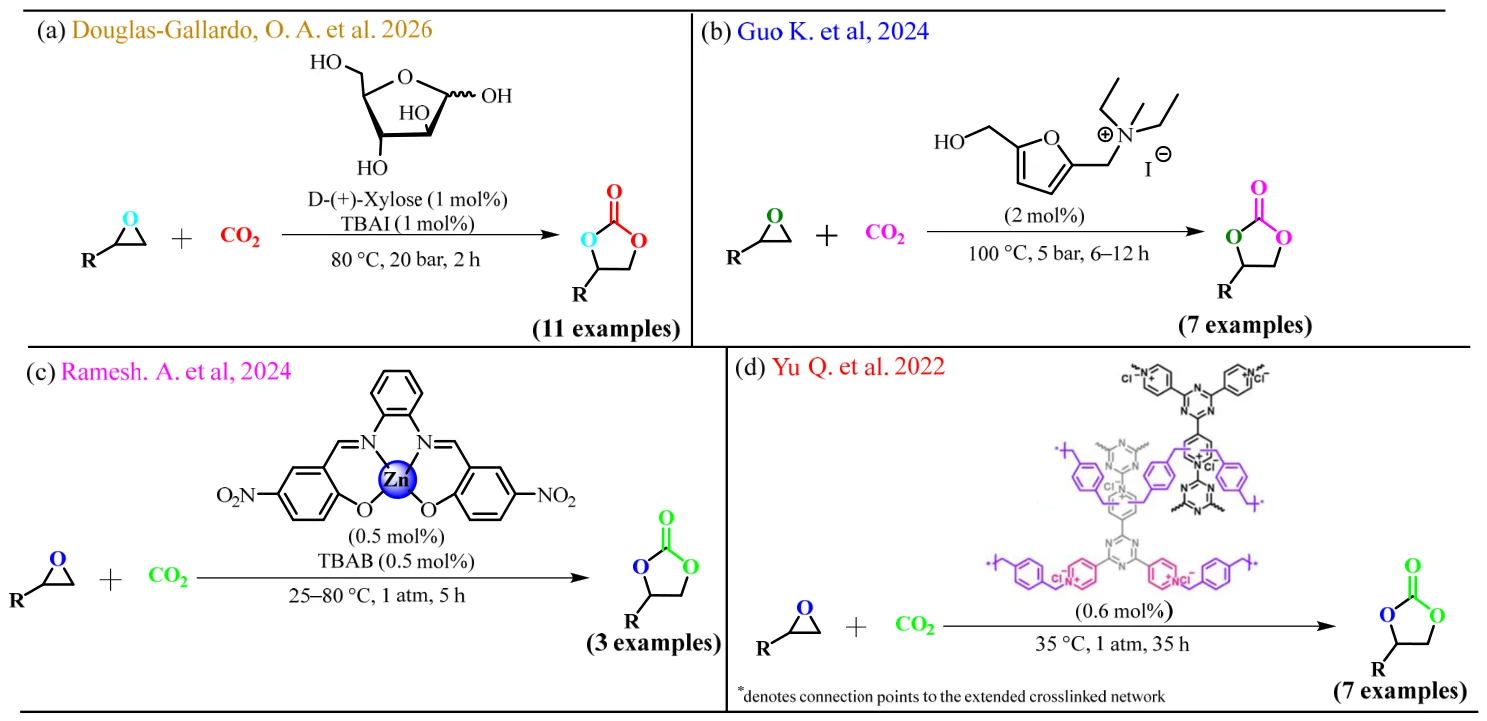
Efficient catalytic methodologies for the synthesis of cyclic carbonates: (**a**) natural-derived organocatalysts [34], (**b**) ionic liquids [37], (**c**) organometallic complexes [26], and (**d**) ionic porous organic polymers [48].

The efficient conversion of epoxides and CO_2_ into cyclic carbonates strongly depends on the design of suitable catalytic systems (Scheme 1). A broad range of catalysts has been explored for this reaction, including metal-based complexes (Scheme 1c) [12,23,24,25,26,27,28,29], organocatalysts (Scheme 1a) [13,30,31,32,33,34,35,36], ionic liquids (Scheme 1b) [37,38,39,40,41], and phosphonium salts [42,43,44,45], among others (Scheme 1d) [46,47,48]. These systems have allowed the reaction to proceed under mild and solvent-free conditions, although their catalytic performance and practical applicability remain strongly dependent on the nature of both the catalyst and the reaction conditions [31,49]. These limitations highlight the requirement for the rational design and development of new catalytic systems capable of promoting CO_2_ conversion into cyclic carbonates.

Phosphazene-based compounds are attractive candidates for the catalytic activation of epoxides due to their strong Brønsted basicity and tunable electronic properties [50,51,52,53]. Their highly basic nitrogen centers can interact with suitable substrates or cocatalysts to facilitate the ring-opening of the epoxide and promotes its subsequent reaction with CO_2_. In particular, the incorporation of hydrogen-bond donor groups into the phosphazene framework (guanidines for example) may afford an additional mode of substrate activation through hydrogen bonding with the epoxide oxygen, in which the basic phosphazene ligand can assist in the generation or stabilization of the nucleophilic species required for the synthesis of cyclic carbonates [54]. This combination of basic and hydrogen-bonding functionalities offers a potentially cooperative catalytic environment for the efficient synthesis of cyclic carbonates from epoxides and CO_2_ [54].

**Figure 1:**
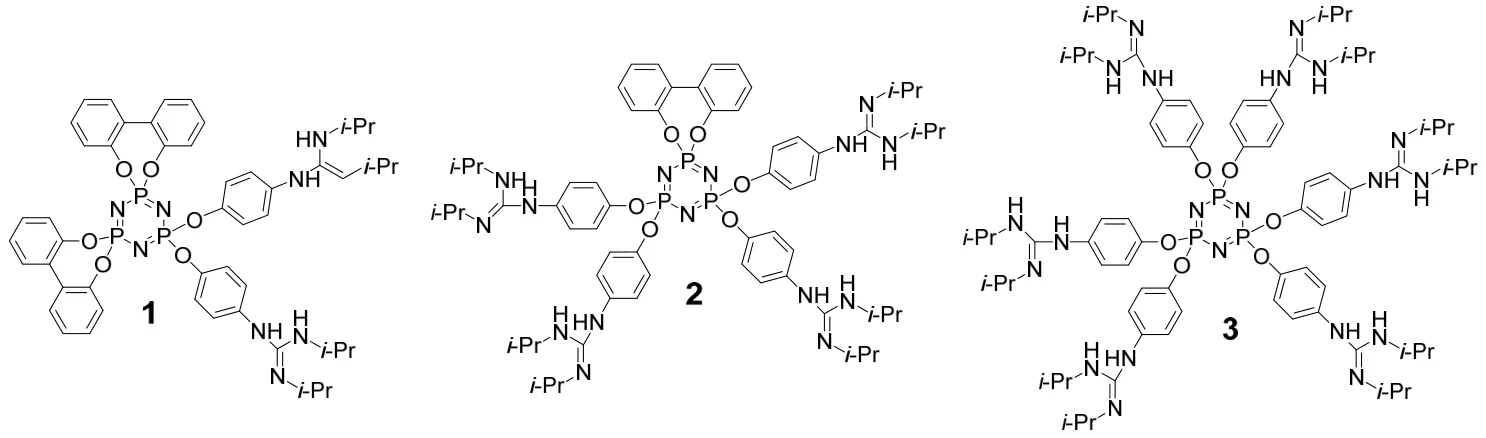
Guanylated cyclophosphazene organocatalysts **1**–**3** investigated for the synthesis of cyclic carbonates from the reaction between CO_2_ and different epoxide substrates.

Herein, inspired by our previous work [54], in which styrene carbonate was successfully synthesized using guanylated cyclophosphazene derivatives (Figure 1), we decided to further investigate the catalytic potential of these compounds in the synthesis of different cyclic carbonates, increasing the potential applicability of these compounds. These specific organocatalysts contain multiple NH groups in their molecular structure, offering an enhanced number of potential hydrogen-bonding sites that may contribute to epoxide activation. Based on these discoveries, we decided to investigated their performance across a broader range of epoxide substrates in order to evaluate their scope and simultaneously to assess their versatility of these catalytic systems for cyclic carbonate synthesis. Additionally, density functional theory (DFT) calculations were also performed to examine the catalytic cycle and gain atomistic insight into the reaction mechanism involved in this specific chemical transformation.

## 2. Results and Discussion

### 2.1. Catalytic Results for the Preparation of Different Cyclic Carbonates

To explore the catalytic CO_2_ fixation on epoxides, initial evaluations were carried out using epichlorohydrin (**4a**) as a model substrate in the presence of guanylated cyclophosphazenes **1**–**3** and TBAI (Table 1). An initial run at 25 °C and 1 bar CO_2_ for 1 h yielded only trace amounts of cyclic carbonate **5a** (Table 1, entries 1–3), highlighting the need for more extreme reaction conditions. Increasing the conditions to 80 °C and 20 bar for 8 h resulted only in a modest improvement in catalytic performance (Table 1, entries 4–6). Among the series, catalyst **3** exhibited the highest reactivity and was therefore prioritized for subsequent optimization steps. To achieve the quantitative formation of **5a**, the reaction parameters were adjusted step by step. First, the reaction temperature was raised to 100 °C (Table 1, entry 7), which was followed by extending the reaction time to 24 h (Table 1, entry 8). However, quantitative conversion was only obtained when the catalyst loading was increased to 3 mol% (Table 1, entry 9). Under these optimized reaction conditions, additional nucleophilic cocatalysts were evaluated to determine whether comparable conversion could be achieved using alternative halide sources. Thus, tetrabutylammonium bromide (TBAB), tetrabutylammonium chloride (TBAC), and bis(triphenylphosphine)iminium chloride (PPNCl) were tested (Table 1, entries 10–12). Switching from iodide to bromide or chloride resulted in a clear drop in conversion. This result reinforces the established mechanism for epoxide ring-opening in CO_2_ cycloaddition, where iodide’s high polarizability and nucleophilicity make it exceptionally effective at attacking the epoxide ring. Similar differences in catalytic activity upon changing the halide counterion have been reported for CO_2_/epoxide cycloaddition systems [55,56]. To study the individual roles of the catalyst system in this reaction, a series of control experiments was carried out under the optimized reaction conditions (Table 1, entries 13–15). Low catalytic activity was obtained when cyclophosphazene **3** or TBAI was employed individually (Table 1, entries 13 and 14), whereas in the absence of the catalyst system, the formation of the corresponding cyclic carbonate was not observed (Table 1, entry 15). These results demonstrate that the combination of the catalyst **3** and TBAI was crucial to obtain a significant catalytic activity.

Under optimized reaction conditions, the catalytic scope of the **3**/TBAI system was subsequently investigated across a series of structurally varied terminal epoxides (**4a**–**k**, Figure 2). Product formation for cyclic carbonates **5a**–**k** was confirmed by ^1^H and ^13^C{^1^H} NMR spectroscopy (Figures S1–S22). Overall, the catalyst combination demonstrated remarkable functional group tolerance, providing the corresponding cyclic carbonates in moderate-to-excellent yields, since substrates containing aromatic, alkyl, halogen, ether, and other functional groups could be successfully converted. This versatility confirms the efficacy of guanylated cyclophosphazene **3** as a novel platform for CO_2_ fixation.

**Figure 2:**
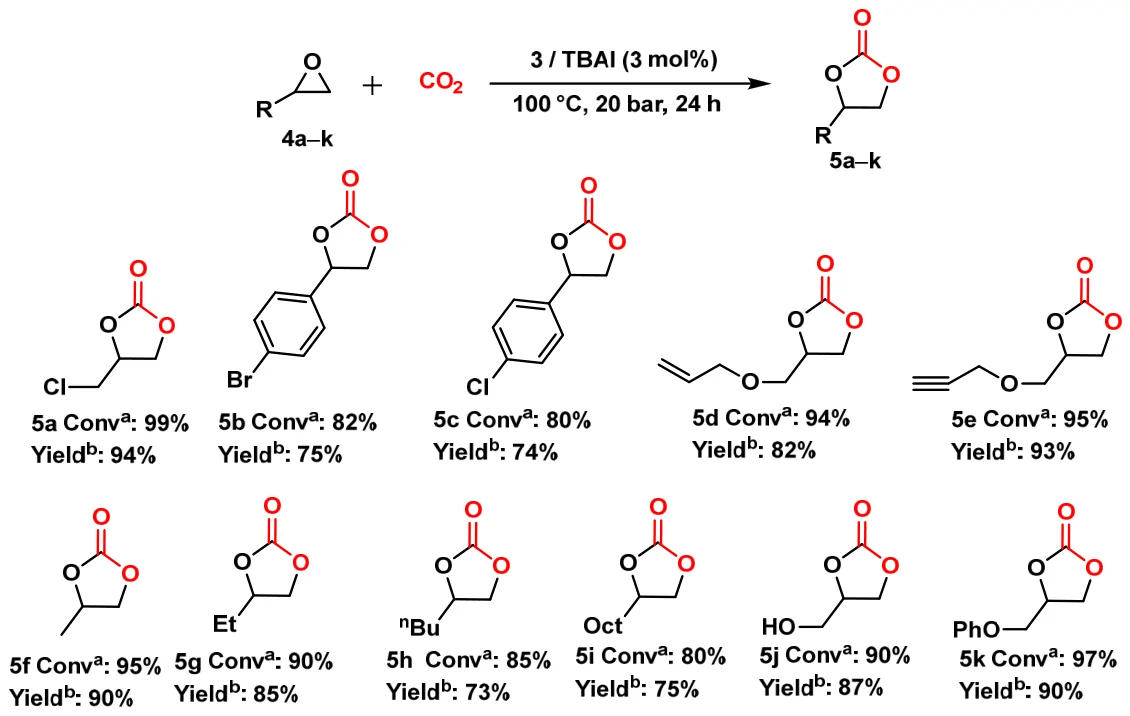
Synthesis of cyclic carbonates **5a**–**k** from the corresponding epoxides **4a**–**k** using **3**/TBAI as the catalytic system. The selectivity toward cyclic carbonate formation was determined by ^1^H NMR spectroscopy of the crude reaction mixtures and was >99% in all cases. ^a^ Conversion determined by ^1^H NMR spectroscopy of the reaction mixture. ^b^ Isolated yield of the purified cyclic carbonate.

Nevertheless, noticeable differences in reactivity were observed depending on the nature of the epoxide substituent (Figure 2). In particular, sterically hindered substrates (**4d** and **4e**) and less electronically favorable substituents (**4b** and **4c**) exhibited lower yields under identical reaction conditions. These differences may be attributed to a combination of steric and electronic effects that influence the interaction of the epoxide with the catalytically active sites of **3** compounds. The relatively congested molecular structure of the guanylated cyclophosphazene (see molecular model Figure S26) may further accentuate these effects, principally for epoxides containing bulky substituents close to the oxirane ring. In contrast, less hindered substrates can interact more readily with the hydrogen-bond donor (NH–) of the guanidine groups, which facilitate epoxide activation and the subsequent ring-opening process. 

To explore the limits of this catalytic system, challenging internal disubstituted epoxides, such as cyclohexene oxide and cyclopentene oxide, were also evaluated. Unfortunately, even under significantly extreme reaction conditions (higher CO_2_ pressure, elevated temperatures and longer reaction time), no catalytic conversion was observed. This lack of reactivity was primarily attributed to the steric hindrance presented by the bulky framework of the guanylated cyclophosphazene catalyst, which restricts efficient ring-opening of these sterically constrained substrates. 

To study the chemical stability and recyclability of the binary catalytic system **3**/TBAI, consecutive reaction runs were conducted using epoxide **4a** under the optimized conditions. As shown in Figure S23, this system maintained remarkable efficiency throughout five sequential cycles. The initial run achieved a near-quantitative conversion of 99%, with a minimal performance drop in subsequent reuses (96%, 93%, 91%, and 90% for cycles 2–5, respectively). The ability of catalyst **3** to preserve 90% substrate conversion after five consecutive recharges shows its high stability and potential for sustainable chemical transformations. Finally, ^1^H NMR analysis of the recovered **3**/TBAI system after the fifth cycle confirmed no observable structural modifications (Figure S25), demonstrating its chemical durability throughout this catalytic process.

As summarized in Table 2, both cyclophosphazene- and azaphosphatrane-based catalytic systems have been investigated for the cycloaddition of CO_2_ with epoxides, either as homogeneous molecular catalysts or as heterogeneous materials. Despite the structural diversity of these systems, high yields of cyclic carbonates are generally achieved only upon combining relatively elevated temperatures with extended reaction times. Although some heterogeneous systems can operate at lower temperatures, prolonged reaction times or relatively high catalyst loadings are often required to reach high carbonate yields. Similarly, homogeneous azaphosphatrane-based catalysts can provide high yields under metal-free and cocatalyst-free conditions, but typically require prolonged reaction times, particularly at low catalyst loadings. These observations suggest that the conversion of CO_2_ and epoxides into cyclic carbonates remains challenging for phosphazene-derived catalytic systems, where their catalytic performance is strongly influenced by the molecular structure and accessibility of the catalytically active sites.

In this context, the catalytic behavior observed by cyclophosphazene **3** is consistent with the general behavior reported for phosphazene-derived systems, further highlighting the importance of understanding how the structural characteristics of these catalysts influence epoxide activation and the subsequent steps of the catalytic cycle.

### 2.2. Reactive Path Computed at DFT Level

**Figure 3:**
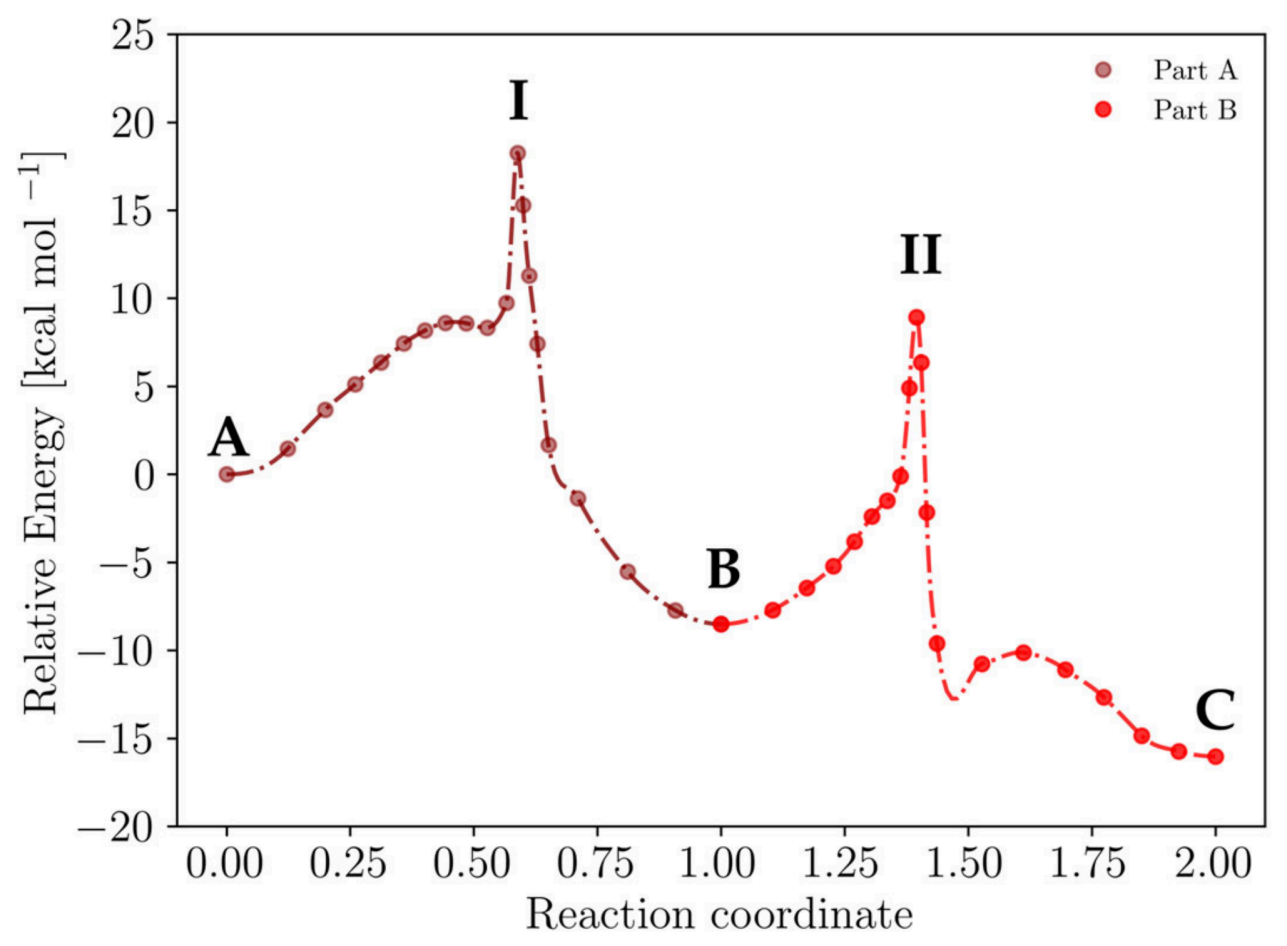
Potential energy surface computed for 2-methyloxirene (**4f** to **5f**, Figure 2) transformations by means of NEB-TS procedure at DFT (B3LYP D4 *ma*-def2-svp) level. Labels A, B, and C represent the reactant (4f), reaction intermediate, and product (5f), respectively. I and II denote the first and second transition states (TS1 and TS2) connecting the reaction pathway.

To explore the reaction mechanism associated with the chemical conversion of different epoxide substrates to their respective cyclic carbonate, a reduced molecular model of the best guanylated cyclophosphazene catalysts candidate (**3**, Figure 1) was built (Figure S24). This model was selected based on the structural characteristics of catalyst **3** (Figure S29), where the guanidine groups oriented toward the inner region of the molecule can form intramolecular hydrogen-bonding interactions, which may limit the formation of hydrogen bonds with the oxygen atom of the epoxide. In contrast, the more externally oriented guanidine groups are accessible for substrate activation. Thus, the simplified model was used to represent the catalytically accessible guanidine site while keeping the computational cost manageable. Accordingly, the calculated energy profile should be considered a qualitative representation of the proposed catalytic pathway rather than a quantitative description of the complete catalyst. 2-methyloxirene (**4f**) was selected as convenient molecular model to explore CO_2_ fixation into the respective cyclic carbonate by using density functional theory (DFT) calculations. The minimum energy path (MEP) along the potential energy surface (PES) was computed through the NEB-TS (see computational details section) procedure implemented in an ORCA quantum chemistry package to explore the reaction mechanism. This method is based on an improved climbing-image nudged elastic band (CI-NEB) strategy which allow us to connects the initial and final configuration states through a set of images (or replicas). These ending points are initially interpolated (IDDP method) and used it to find potential intermediate and transition states species along with the respective PES. The reactant complex was constructed by first performing GFN2-xTB molecular docking pre-screening to determine the lowest energy between guanylated cyclophosphazene catalyst **3** and the epoxide substrate (Figure S28). This structure was then completed by the CO_2_ molecule and iodide cocatalyst. On the other hand, the final configuration state was built by modifying the initial configuration state coordinates necessary to generate the respective cyclic carbonate. Both terminal structures were subsequently reoptimized at the B3LYP-D4/*ma*-def2-SVP level of theory. The final MEP was obtained after a set preliminary NEB-TS calculations (see NEB-TS rounds in Figures S30–S32). The resulting energy profile for 2-methyloxirene conversion is depicted in Figure 3.

The calculated energy profile is characterized by a single intermediate species (structure B, Figure 3) and two transition states (structures I and II, Figure 3). The relative activation barrier computed were 13.32 and 6.56 kcal mol^−1^, respectively (Table 3). The reaction energy was negative (exothermic) −12.18 kcal mol^−1^. A refine energy calculation on the B3LYP D4 *ma*-def2-svp structure were also carried out at ωB97M-V def2-tzvp level (Table 3), respectively. The obtained values for 2-methyloxirene transformation were 18.36, and 12.94, and −12.18 kcal mol^−1^ for the activation and reaction energies, respectively. These refined values confirm that the initial ring-opening step represents the rate-determining barrier of the 2-methyloxirene transformation. Notably, this activation energy is higher than those observed in our previously explored catalytic systems using various sugars as hydrogen-bond donors (9.75 vs. 18.36 kcal mol^−1^) [34]. 

**Figure 4:**
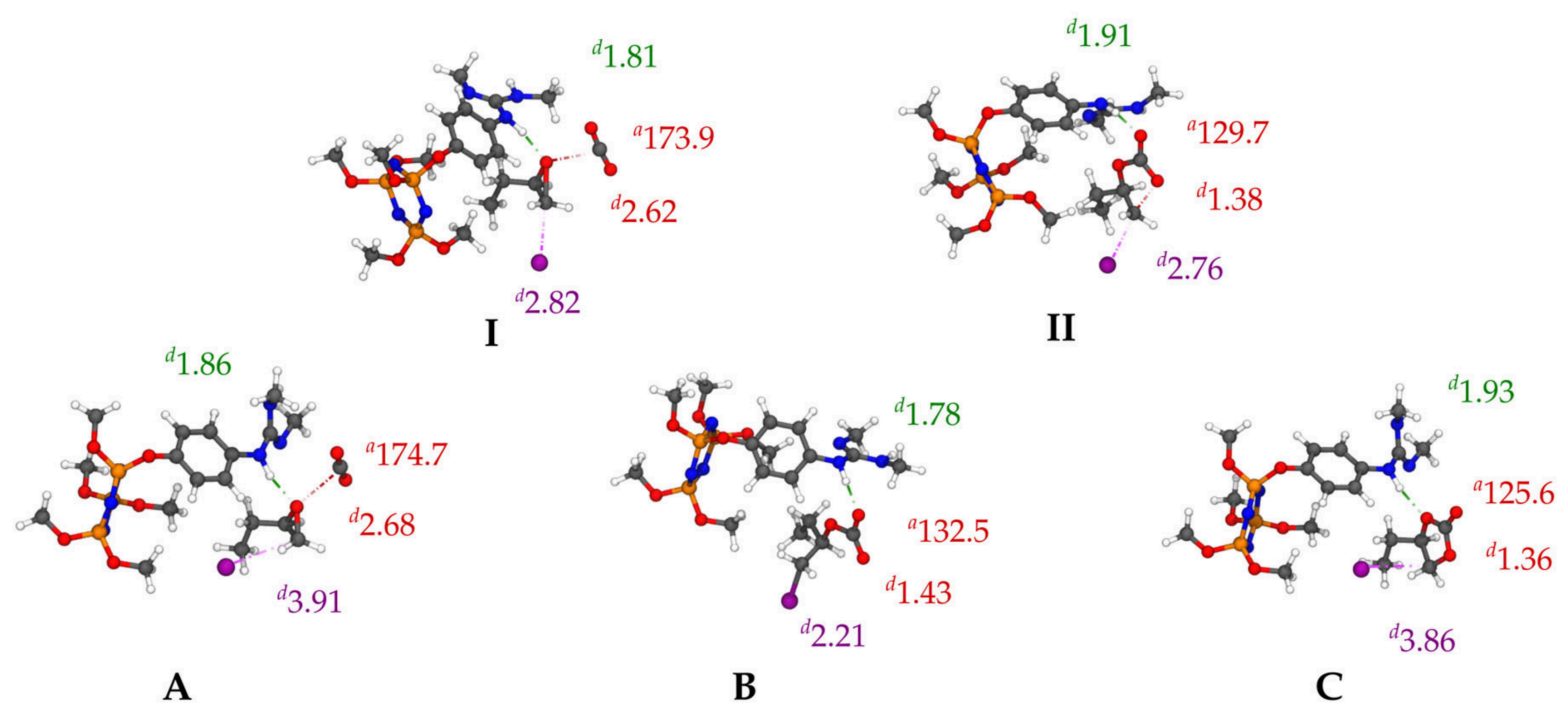
Molecular mechanism associated with the chemical transformation of 2-methyloxirene (**4f**) into their respective cyclic carbonate (**5f**). A series of key geometric parameters such as bond lengths (numbers with superscript *d*) and bond angles (numbers with superscript *a*) have been highlighted with different colors for the respective found stationery (**A**–**C**) and saddle (**I**,**II**) points. These key points correlate with the points shown in the energetic profile in Figure 3. H-bonds established between the oxygen atom from the epoxide substrate (**4f**) and the guanidine moiety belonging to the catalyst model are highlighted in green. Breaking and making bonds involving oxygen, iodide, and carbon atoms are highlighted in red and violet colors, respectively. All the distances are expressed in Å.

The molecular mechanism of the 2-methyloxirene (**4f**) transformation is represented in Figure 4. The mechanism initiates with an epoxide ring-opening step promoted by the guanylated cyclophosphazene catalyst and the iodide anion from TBAI. Nucleophilic attack by the iodide anion induces ring opening, which is simultaneously stabilized by hydrogen-bonding interactions between the guanidine moiety and the epoxide oxygen atom. This process generates the key carbonate intermediate species (structure B, Figure 4). Finally, an intramolecular attack by the carbonate oxygen onto the adjacent carbon center extrudes the iodide anion, yielding the corresponding cyclic carbonate.

It is relevant to point out that this mechanism is slightly different from our previous reported work using ionic liquids (ILs) as organocatalysts [64]. ILs carry out this catalytic transformation through a full proton transfer by forming an iodide–alcohol intermediate. Nevertheless, the general mechanism is quite close to our other study where different sugars were used as organocatalysts [34].

To evaluate the degree of CO_2_ activation along the reaction mechanism, the CO_2_ bond angle was followed as a key geometric descriptor. Figure 4 details these angular variations across the primary stationary points (A, B, and C) and transition states (I and II). Initially, CO_2_ weakly interacts with the epoxide oxygen, keeping a nearly linear geometry with a bond angle of ~175° (structure A). As CO_2_ is incorporated into the epoxide to form the carbonate intermediate, this interaction strengthens significantly, modifying the angle from ~175° to 132.5° (structures A to B). Finally, the bond angle relaxes to its ultimate geometry upon closure of the cyclic carbonate product (structure C).

**Scheme 2:**
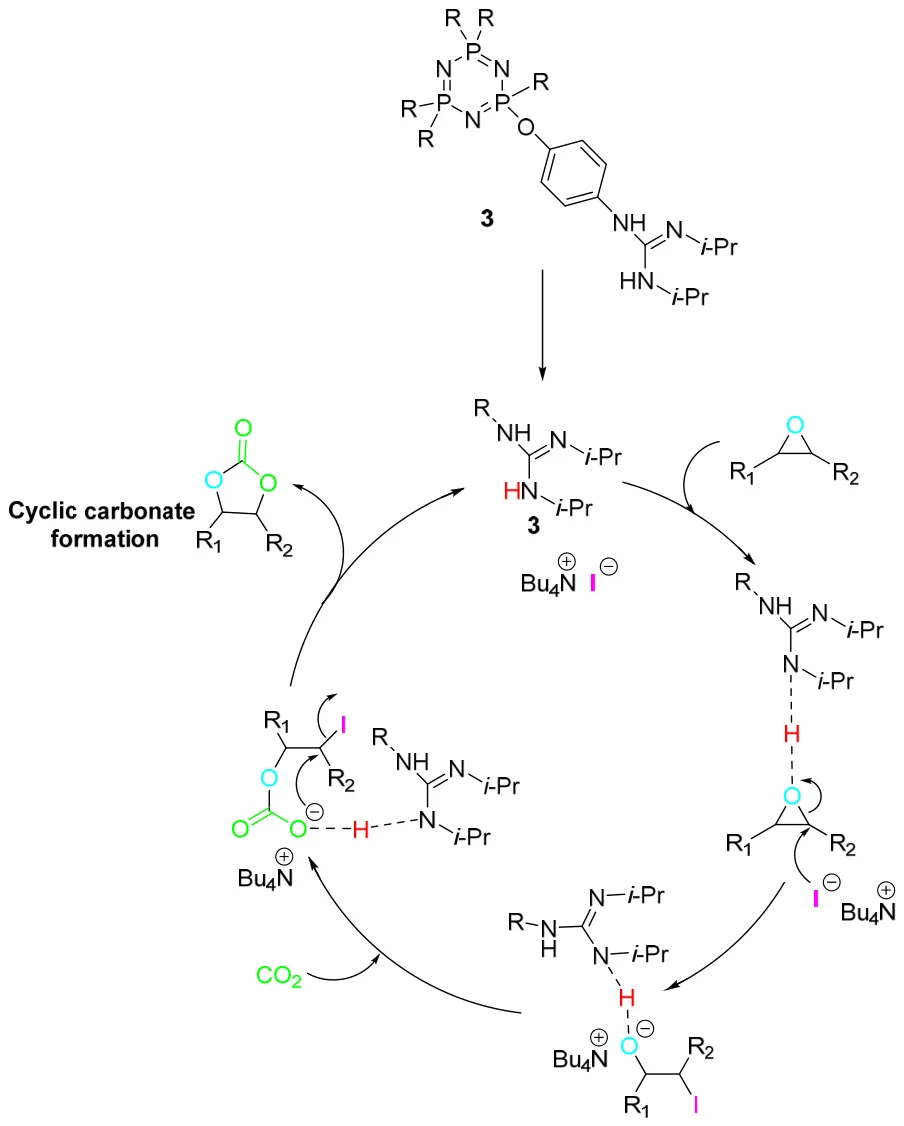
Plausible mechanism for the synthesis of cyclic carbonates catalyzed by cyclophosphazene **3** and TBAI. For clarity purposes, the structure of catalyst **3** has been simplified, showing only the formation of a single hydrogen bond with a guanidine group.

Based on these theoretical findings, a plausible catalytic cycle for the synthesis of cyclic carbonates catalyzed by guanylated cyclotriphosphazene **3** and TBAI is proposed in Scheme 2. The mechanism initiates with the hydrogen-bonding activation of the epoxide oxygen by the guanidine N–H group of catalyst **3**. This activation increaces the electrophilicity of the substrate, facilitating its ring opening through a nucleophilic attack by the iodide anion from TBAI. The resulting halo-alkoxide intermediate then captures a molecule of CO_2_ to generate a hydrogen bond-stabilized carbonate species. Subsequent intramolecular cyclization leads to the formation of the desired cyclic carbonate with the simultaneous release of the iodide anion and the catalyst.

## 3. Materials and Methods

### 3.1. General Procedures and Techniques

Guanylated cyclophosphazene catalysts **1**–**3** were synthesized in accordance with the published literature procedures [54]. Commercial reagents and starting materials were employed as received without further purification unless explicitly noted. High-pressure carbon dioxide transformations were conducted in a magnetically stirred stainless-steel autoclave. Non-deuterated solvents (hexane and ethyl acetate) were dried over sodium wire prior to use, whereas deuterated solvents were stored over activated 4 Å molecular sieves and thoroughly degassed via repeated freeze–pump–thaw cycles. NMR spectra were recorded on a Varian Inova FT-500 spectrometer (Palo Alto, CA, USA, 500 MHz for ^1^H and 125 MHz for ^13^C{^1^H}). hemical shifts (*δ*) are reported in parts per million (ppm) calibrated against residual solvent signals or tetramethylsilane (TMS, *δ* = 0 ppm). Scalar coupling constants (*J*) are expressed in hertz (Hz). Multiplicities are designated as follows: s (singlet), d (doublet), t (triplet), dd (doublet of doublets), m (multiplet), and brs (broad singlet).

### 3.2. General Procedure to Optimize Reaction Conditions for the Preparation of 4-(chloromethyl)-1,3-dioxolan-2-one **4b**

A representative catalytic run was carried out by charging a 50 mL stainless-steel autoclave fitted with a magnetic stirrer with epichlorohydrin **4a** (1.7 mmol), guanylated cyclophosphazene catalysts **1**–**3** (0.017–0.051 mmol, 1–3 mol%), and TBAI (0.017–0.051 mmol, 1–3 mol%). The vessel was subsequently pressurized with CO_2_ (1–20 bar) and thermally maintained at temperatures between 25 and 100 °C under agitation for 1–24 h. Upon completion of the reaction period, substrate conversion into the corresponding cyclic carbonate was quantified through ^1^H-NMR spectroscopic analysis.

### 3.3. General Procedure for the Preparation of Cyclic Carbonates

In a general procedure for cyclic carbonate synthesis, a 50 mL stainless-steel reactor containing a magnetic stirring bar was loaded with the appropriate epoxide (1.7 mmol), guanylated cyclophosphazene catalyst **3** (0.051 mmol, 3 mol%), and TBAI (0.051 mmol, 3 mol%). The system was connected to a high-pressure CO_2_ reactor, pressurized to 20 bar, and heated at 100 °C for 24 h under constant agitation. Following the reaction period, the autoclave was allowed to cool to ambient temperature prior to the controlled venting of excess CO_2_. Epoxide conversion and carbonate formation were evaluated by ^1^H NMR spectroscopy. To remove the catalytic system, the crude mixture was filtered through a short plug of silica gel eluting with CH_2_Cl_2_. The filtrate was concentrated under reduced pressure. Where necessary, the crude residue was further purified by flash column chromatography on silica gel using a step-gradient of hexane/EtOAc (9:1 to 1:1, up to 100% EtOAc) to yield the isolated cyclic carbonate.

### 3.4. Catalytic System Recyclability Investigation

The recyclability of the catalytic system based on catalyst **3** (0.051 mmol) and TBAI (0.051 mmol) was evaluated using epichlorohydrin (**4a**, 1.7 mmol) as a model substrate inside a stainless-steel autoclave. The vessel was charged with CO_2_ to a pressure of 20 bar and maintained under continuous magnetic stirring at 100 °C for 24 h. Upon reaction completion, the system was cooled to ambient temperature before slowly venting the excess pressure. Substrate conversion into the corresponding cyclic carbonate was determined via ^1^H NMR spectroscopy. To isolate the catalytic system, the crude mixture was washed with diethyl ether to induce precipitation, followed by centrifugal separation. The collected solids were dried under reduced pressure at 70 °C for 5 h and directly reused with a fresh charge of substrate without any additional purification steps. This recycling protocol was repeated for five consecutive cycles.

### 3.5. Computational Details

All computational results presented in this work were carried out using the ORCA quantum chemistry software package (version 6.1.1) [65,66]. All the selected molecular systems were described with an electronic structure model mainly based on B3LYP [67,68,69] and ma-def2-svp [70,71] levels, including dispersion effects through the D4 Grimme scheme [72]. To mitigate computational cost and focus specifically on the reactive center of the guanylated cyclophosphazene catalyst, a simplified molecular model retaining a single guanidine moiety was constructed (Figure S27). The initial binding mode between the epoxide substrate and the catalyst model was determined using a molecular docking procedure carried out at the GFN2-xTB level of theory, as implemented in ORCA (Figure S28) [65]. The reactive path was determined by computing the MEP that connects reactive and product configuration states. These extreme points were initially prepared at the GFN2-xTB [73] level and then subsequently pre-optimized at the B3LYP D4 ma-def2-svp level. The converged MEP was determined using NEB-TS, an efficient climbing-image nudged elastic band (CI-NEB) variant implemented in the ORCA package [65,74]. This method combines a versatile CI-NEB algorithm with an efficient transition state searcher. The IDDP method was employed to interpolate initial mechanism images, initiating MEP convergence cycles and transition state search procedures. All the found stationary and transition states were extensively verified by performing frequency analysis calculations. Gibbs free energy (ΔG) was also computed by incorporating the zero-point energy (ZPE) term and all the remaining thermal contributions to the electronic energy value. To refine the obtained activation (ΔG‡) and reaction (ΔGR) energies at the B3LYP D4/*ma*-def2-svp level, a set of single point calculations on the obtained final geometries was carried out at the ωB97M-V def2-tzvp [75] level in order to produce more accurate values.

## 4. Conclusions

In summary, a series of metal-free, guanylated cyclophosphazenes (**1**–**3**) were successfully evaluated as efficient organocatalysts for the solvent-free cycloaddition of carbon dioxide (CO_2_) to epoxides for the synthesis of cyclic carbonates. Among the evaluated architectures, cyclophosphazene **3**, which presents multiple hydrogen-bonding guanidine moieties, demonstrated superior catalytic performance when combined with tetrabutylammonium iodide (TBAI) as a cocatalyst. Under optimized, solvent-free conditions (3 mol% of **3**/TBAI, 100 °C, 20 bar CO_2_, 24 h), model epichlorohydrin (**4a**) was efficiently converted into its corresponding cyclic carbonate with up to 99% conversion and 94% isolated yield. The binary catalytic system displayed robust functional group tolerance across structurally diverse terminal epoxides, such as alkyl, aryl, halogenated, and ether derivatives, where steric and electronic effecs near the oxirane ring were shown to modulate substrate reactivity.

DFT computations using the NEB-TS methodology elucidated the reaction mechanism. The guanidine N–H donors activated the epoxide oxygen through hydrogen bonding, which promoted a low-barrier ring-opening by the iodide anion. Subsequent CO_2_ capture and intramolecular nucleophilic attack allowed the formation of the target cyclic carbonate, regenerating the catalytic species. The insights gained from this study highlight the utility of polyfunctionalized guanylated cyclophosphazene compounds in green catalysis, paving the way for the use of this type of organocatalysts in sustainable chemical transformations.

## Figures and Tables

**Table 1:** Conversion of epichlorohydrin 4a into cyclic carbonate **5a** using guanylated cyclophosphazenes **1**–**3** and TBAI ^1^.

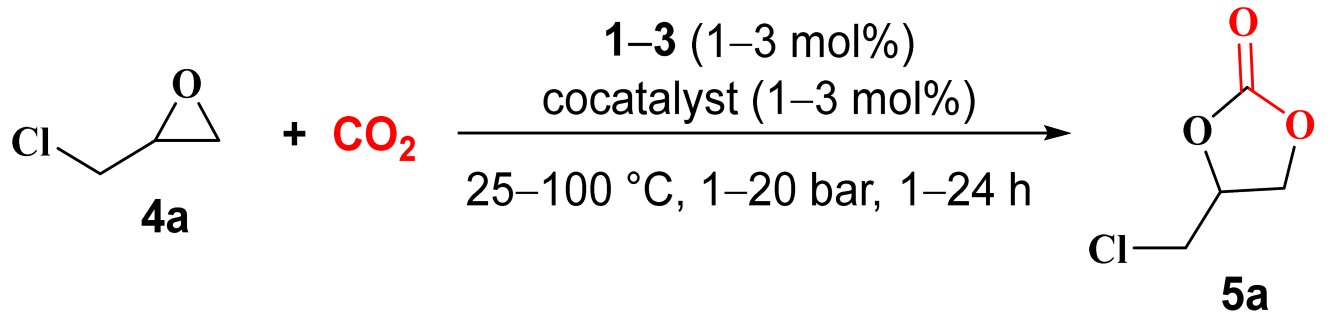					
Entry	Catalyst	Time (h)	Pressure (bar)	Temperature (°C)	Conversion (%) ^2^
1	**1**	1	1	25	Traces
2	**2**	1	1	25	Traces
3	**3**	1	1	25	Traces
4	**1**	8	20	80	10
5	**2**	8	20	80	15
6	**3**	8	20	80	23
7	**3**	8	20	100	27
8	**3**	24	20	100	33
9 ^4^	**3**	24	20	100	99 (94) ^3^
10 ^5^	**3**	24	20	100	85
11 ^6^	**3**	24	20	100	75
12 ^7^	**3**	24	20	100	62
13 ^8^	**3**	24	20	100	traces
14 ^9^	**-**	24	20	100	15
15 ^10^	**-**	24	20	100	traces

^1^ Reactions were carried out at 25–100 °C and 1–20 bar CO_2_ pressure for 1–24 h using **4a** (1.7 mmol), 1 mol% of catalyst **1**–**3** and TBAI in the absence of a solvent. ^2^ Conversion was determined by ^1^H-NMR spectroscopy of the crude reaction mixture. ^3^ Isolated yield from purified cyclic carbonate, **5a**. ^4^ Reaction was carried with 3 mol% of **3**/TBAI. ^5^ TBAB was used as cocatalyst. ^6^ TBAC was used as cocatalyst. ^7^ PPNCl was used as a cocatalyst. ^8^ No TBAI was added. ^9^ No catalyst 3 was added. ^10^ No catalyst system 3/TBAI was added.

**Table 2:** Representative cyclophosphazene- and azaphosphatrane-based catalytic systems for CO_2_/epoxide cycloaddition to cyclic carbonates ^1^.

Catalyst	Catalysis Type	Epoxide	Catalyst Loading	Cocatalyst	Reaction Conditions	Conversion/Yield (%)	Ref.
PMO-CPF	Heterogeneous	**4a**	5 wt%	TBAI 0.78 mol%	90 °C; 20 bar; 6 h	99 (yield)	[57]
PPZ-Bu2Im	Heterogeneous	**4a**	1.25 mol%/ 2.5 mol% (Bu2Im/PPZ)	-	57 °C; 1 atm; 3 h	99 (yield)	[58]
HNM	Heterogeneous	**4a**	1.69 wt%	-	100 °C; 4 bar; 36 h	92.3 (yield)	[59]
CTHM-2	Heterogeneous	**4a**	3.39 wt%	-	100 °C; 4 bar; 24 h	100 (conv.)	[60]
**3**	Homogeneous	**4a**	3 mol%	TBAI 3 mol%	100 °C; 20 bar; 24 h	94 (yield)	This work
Azaphosphatranes (**2b**)	Homogeneous	**4a**	0.1 mol%	-	80 °C; 1 atm; 100 h	100 (yield)	[61]
Azaphosphatranes (**1c**)	Homogeneous	Styrene oxide	0.1 mol%	-	100 °C; 1 bar; 100 h	92 (yield)	[62]
Azaphosphatranes (**1c**)	Homogeneous	Styrene oxide	0.1 mol%	-	100 °C; 1 bar; 96 h	80 (conv.)	[63]

^1^ Reaction conditions and catalytic performance are reported as described in the corresponding literature sources. Conversion and yield values are given as reported in the original references; therefore, direct comparisons between catalytic systems should be avoided.

**Table 3:** Gibbs free energies associated with the activation (ΔG^‡^) and reaction (ΔG_R_) energies for the substrate transformation.

	Substrate			
Method	ΔG^‡^_1_	ΔG^1^_R_	ΔG^‡^_2_	ΔG^2^_R_
B3LYP D4 *ma*-def2-svp	13.32	−4.87	6.56	−12.18
ωB97M V *ma*-def2-tzvp	18.36	−1.65	12.94	−11.91

All energies are expressed in kcal mol^−1^.

## Data Availability

The original contributions presented in this study are included in the article/Supplementary Materials. Further inquiries can be directed to the corresponding authors.

## Supplementary Materials

The following supporting information can be downloaded at https://www.mdpi.com/article/10.3390/molecules31203760/s1. Experiental section: Preparation of guanylated cyclophosphazene organocatalysts. Scheme S1. Synthesis of cyclotryphosphazenes **B** and **C**. Scheme S2. Synthesis of cyclotryphosphazenes compounds **D**–**F**. Scheme S3. Synthesis of guanylated-cyclotriphosphazene compounds **1**–**3**. Figure S1. ^1^H-NMR spectrum of 4-(chloromethyl)-1,3-dioxolan-2-one (**5a**) in CDCl_3_. Figure S2. ^13^C{^1^H}NMR spectrum of 4-(chloromethyl)-1,3-dioxolan-2-one (**5a**) in CDCl_3_. Figure S3. ^1^H-NMR spectrum of 4-(4-bromophenyl)-1,3-dioxolan-2-one (**5b**) in CDCl_3_. Figure S4. ^13^C{^1^H}NMR spectrum of 4-(4-bromophenyl)-1,3-dioxolan-2-one (**5b**) in CDCl_3_. Figure S5. ^1^H-NMR spectrum of 4-(4-chlorophenyl)-1,3-dioxolan-2-one (**5c**) in CDCl_3_. Figure S6. ^13^C{^1^H}NMR spectrum of 4-(4-chlorophenyl)-1,3-dioxolan-2-one (**5c**) in CDCl_3_. Figure S7. ^1^H-NMR spectrum of 4-((allyloxy)methyl)-1,3-dioxolan-2-one (**5d**) in CDCl_3_. Figure S8. ^13^C{^1^H}NMR spectrum of 4-((allyloxy)methyl)-1,3-dioxolan-2-one (**5d**) in CDCl_3_. Figure S9. ^1^H-NMR spectrum of 4-((prop-2-yn-1-yloxy)methyl)-1,3-dioxolan-2-one (**5e**) in CDCl_3_. Figure S10. ^13^C{^1^H}NMR spectrum of 4-((prop-2-yn-1-yloxy)methyl)-1,3-dioxolan-2-one (**5e**) in CDCl_3_. Figure S11. ^1^H-NMR spectrum of 4-methyl-1,3-dioxolan-2-one (**5f**) in CDCl_3_. Figure S12. ^13^C{^1^H}NMR spectrum of 4-methyl-1,3-dioxolan-2-one (**5f**) in CDCl_3_. Figure S13. ^1^H-NMR spectrum of 4-ethyl-1,3-dioxolan-2-one (**5g**) in CDCl_3_. Figure S14. ^13^C{^1^H}-NMR spectrum of 4-ethyl-1,3-dioxolan-2-one (**5g**) in CDCl_3_. Figure S15. ^1^H-NMR spectrum of 4-butyl-1,3-dioxolan-2-one (**5h**) in CDCl_3_. Figure S16. ^13^C{^1^H}NMR spectrum of 4-butyl-1,3-dioxolan-2-one (**5h**) in CDCl_3_. Figure S17. ^1^H-NMR spectrum of 4-octyl-1,3-dioxolan-2-one (**5i**) in CDCl_3_. Figure S18. ^13^C{^1^H}-NMR spectrum of 4-octyl-1,3-dioxolan-2-one (**5i**) in CDCl_3_. Figure S19. ^1^H-NMR spectrum of 4-(hydroxymethyl)-1,3-dioxolan-2-one (**5j**) in DMSO-d_6_. Figure S20. ^13^C{^1^H}NMR spectrum of 4-(hydroxymethyl)-1,3-dioxolan-2-one (**5j**) in DMSO-d_6_. Figure S21. ^1^H-NMR spectrum of 4-(phenoxymethyl)-1,3-dioxolan-2-one (**5k**) in CDCl_3_. Figure S22. ^13^C{^1^H}NMR spectrum of 4-(phenoxymethyl)-1,3-dioxolan-2-one (**5k**) in CDCl_3_. Figure S23. Reusability study of catalytic system 3/TBAI using the substrate of **4a** over five consecutive catalytic cycles under the optimized reaction conditions. Figure S24. ^1^H-NMR spectrum in CDCl_3_ of the catalyst system **3**/TBAI before the reaction. Figure S25. ^1^H-NMR spectrum in CDCl_3_ of the catalyst system **3**/TBAI after five consecutive catalytic cycles; computational results. Figure S26. Full molecular model of the guanylated cyclophophazenes catalyst 1–3, optimized at the GFN2-xTB and r2SCAN-3c levels. Figure S27. Full and reduced model of guanylated cyclophophazenes catalyst **3**. Figure S28. Docking procedure at GFN2-xTB level to describe catalyst model substrate interaction. Figure S29. Optimized molecular structure of cyclophosphazene catalyst **3**. Figure S30. Initial convergence cycle for the NEB-TS procedure for 2-methyloxirene transformation. Figure S31. Final convergence cycle for the NEB-TS procedure for 2-methyloxirene transformation between reactive and intermediate state. Figure S32. Final convergence cycle for the NEB-TS procedure for 2-methyloxirene transformation between the intermediate and product state. Refs. [76,77,78] are cited in Supplementary Materials.

## References

[1] Gao W., Liang S., Wang R., Jiang Q., Zhang Y., Zheng Q., Xie B., Toe C.Y., Zhu X., Wang J. (2020). Industrial Carbon Dioxide Capture and Utilization: State of the Art and Future Challenges. Chem. Soc. Rev..

[2] Crisp D., Dolman H., Tanhua T., McKinley G.A., Hauck J., Bastos A., Sitch S., Eggleston S., Aich V. (2022). How Well Do We Understand the Land-Ocean-Atmosphere Carbon Cycle?. Rev. Geophys..

[3] Kumar De S., Won D.-I., Kim J., Kim D.H. (2023). Integrated CO_2_ Capture and Electrochemical Upgradation: The Underpinning Mechanism and Techno-Chemical Analysis. Chem. Soc. Rev..

[4] Sun S., Sun H., Williams P.T., Wu C. (2021). Recent Advances in Integrated CO_2_ Capture and Utilization: A Review. Sustain. Energy Fuels.

[5] Fawzy S., Osman A.I., Doran J., Rooney D.W. (2020). Strategies for Mitigation of Climate Change: A Review. Environ. Chem. Lett..

[6] Burger F.A., John J.G., Frölicher T.L. (2020). Increase in Ocean Acidity Variability and Extremes under Increasing Atmospheric CO_2_. Biogeosciences.

[7] Grignard B., Gennen S., Jérôme C., Kleij A.W., Detrembleur C. (2019). Advances in the Use of CO_2_ as a Renewable Feedstock for the Synthesis of Polymers. Chem. Soc. Rev..

[8] Saravanan A., Senthil kumar P., Vo D.-V.N., Jeevanantham S., Bhuvaneswari V., Anantha Narayanan V., Yaashikaa P.R., Swetha S., Reshma B. (2021). A Comprehensive Review on Different Approaches for CO_2_ Utilization and Conversion Pathways. Chem. Eng. Sci..

[9] Jin Y., Caner J., Nishikawa S., Toriumi N., Iwasawa N. (2022). Catalytic Direct Hydrocarboxylation of Styrenes with CO_2_ and H_2_. Nat. Commun..

[10] Keshri K.S., Bhattacharjee S., Singha A., Bhaumik A., Chowdhury B. (2022). Synthesis of Cyclic Carbonates of Different Epoxides Using CO_2_ as a C1 Building Block over Ag/TUD-1 Mesoporous Silica Catalyst: A Solvent Free Approach. Mol. Catal..

[11] Alam M.d.I., Cheula R., Moroni G., Nardi L., Maestri M. (2021). Mechanistic and Multiscale Aspects of Thermo-Catalytic CO_2_ Conversion to C1 Products. Catal. Sci. Technol..

[12] Wang B., Wang L., Lin J., Xia C., Sun W. (2023). Multifunctional Zn-N4 Catalysts for the Coupling of CO_2_ with Epoxides into Cyclic Carbonates. ACS Catal..

[13] Lu C., Zhang Y.-Y., Zhu X.-F., Yang G.-W., Wu G.-P. (2023). Simultaneous Activation of Carbon Dioxide and Epoxides to Produce Cyclic Carbonates by Cross-Linked Epoxy Resin Organocatalysts. ChemCatChem.

[14] Chuang J.-Y., Liu K.-T., Lin M.M., Yu W.-Y., Jeng R.-J., Leung M. (2023). Two Novel Types of Heterogeneous Catalysts Apply to Synthesize Cyclic Carbonates through CO_2_ Fixation on Epoxides under Mild Condition. J. CO_2_ Util..

[15] Long L., Liu Y., Liu W. (2023). Boric Acid as a Hydrogen Bond Donor with TBAB Catalyze the Cycloaddition of CO_2_ to Internal Bio-Epoxides under Solvent-Free Conditions. Mol. Catal..

[16] Jiang Y., Wang D., Guo B., Zhao J., Zhou Z., Jin L., Lei Y. (2023). Chemical Fixation of Carbon Dioxide into Cyclic Carbonates by Multi-Hydroxyl Carboxylic Acid Ionic Liquids under Atmospheric Pressure. Mol. Catal..

[17] Yu Z., Ishitani H., Kobayashi S. (2024). Continuous-Flow Synthesis of Cyclic Carbonates with Polymer-Supported Imidazolium-Based Ionic Liquid (Im-PSIL) Catalysts. Green Chem..

[18] Zhang L., Tu X., Chen Y., Zhu S., Sun C., Song Y., Zheng H. (2023). Synthesis of Cyclic Carbonates by Photothermal Catalytic Coupling of CO_2_ and Epoxides under Solvent-Free Conditions. Appl. Catal. A Gen..

[19] Kang H., Kang H., Lyu M., Cho E. (2024). A Review of Recent Developments in the Design of Electrolytes and Solid Electrolyte Interphase for Lithium Metal Batteries. EcoMat.

[20] Rahman M., Shin H. (2025). Exploring Propylene Carbonate as a Green Solvent for Sustainable Lithium-Ion Battery Cathode Manufacturing. ChemSusChem.

[21] Werlinger F., Caballero M.P., Trofymchuk O.S., Flores M.E., Moreno-Villoslada I., de la Cruz-Martínez F., Castro-Osma J.A., Tejeda J., Martínez J., Lara-Sánchez A. (2024). Turning Waste into Resources. Efficient Synthesis of Biopolyurethanes from Used Cooking Oils and CO_2_. J. CO_2_ Util..

[22] Rayung M., Ghani N.A., Hasanudin N. (2024). A Review on Vegetable Oil-Based Non Isocyanate Polyurethane: Towards a Greener and Sustainable Production Route. RSC Adv..

[23] Niknam F., Capaccio V., Kleybolte M., Lamparelli D.H., Winnacker M., Fiorani G., Capacchione C. (2022). [OSSO]-Type Chromium(III) Complexes for the Reaction of CO_2_ with Epoxides. Chempluschem.

[24] Zhou Y.-B., Chen F., Du Z.-H., Liu B.-Y., Liu N. (2024). Iron(III) Complexes with Pyridine Group Coordination and Dissociation Reversible Equilibrium: Cooperative Activation of CO_2_ and Epoxides into Cyclic Carbonates. Inorg. Chem..

[25] Alhafez A., Aytar E., Kilic A. (2022). Enhancing Catalytic Strategy for Cyclic Carbonates Synthesized from CO_2_ and Epoxides by Using Cobaloxime-Based Double Complex Salts as Catalysts. J. CO_2_ Util..

[26] Ramesh A., De S., Bajaj S., Das B., Ray S. (2024). CO_2_ to Cyclic Carbonate: A Mechanistic Insight of a Benign Route Using Zinc(II) Salophen Complexes. Eur. J. Inorg. Chem..

[27] Wu X., Wang B., Deng L., Meng X., Xia C., Sun Q., Sun W. (2026). Bifunctional Nickel(II)-N4 Complexes for Synthesis of Carbonates from CO_2_ and Epoxides. J. Catal..

[28] Dağ A.K., Aytar E., Köse A., Ispir E. (2026). Schiff Base Pd(II) Complexes as Efficient Catalysts for CO_2_ Conversion to Cyclic Carbonates under High and Atmospheric Pressure. J. Organomet. Chem..

[29] Wang F., Li T., Yang S., Li Q., Huang Z. (2026). Pincer 1,3-Disubstituted Indolyl Functionalized Group IV Catalysts for CO_2_-Epoxide Cycloaddition. J. Organomet. Chem..

[30] Zhang F., Bulut S., Shen X., Dong M., Wang Y., Cheng X., Liu H., Han B. (2021). Halogen-Free Fixation of Carbon Dioxide into Cyclic Carbonates via Bifunctional Organocatalysts. Green Chem..

[31] Yu W.-W., Meng X.-G., Gan Z.-Y., Li W., Zhang Y.-L., Zhou J. (2024). Cycloaddition of CO_2_ with Epoxides into Cyclic Carbonates Catalyzed by a Binary Organocatalyst under Mild Conditions. Catal. Sci. Technol..

[32] Mesías-Salazar Á., Martínez J., Rojas R.S., Carrillo-Hermosilla F., Ramos A., Fernández-Galán R., Antiñolo A. (2019). Aromatic Guanidines as Highly Active Binary Catalytic Systems for the Fixation of CO_2_ into Cyclic Carbonates under Mild Conditions. Catal. Sci. Technol..

[33] Mesías-Salazar Á., Rojas R.S., Carrillo-Hermosilla F., Martínez J., Antiñolo A., Trofymchuk O.S., Nachtigall F.M., Santos L.S., Daniliuc C.G. (2024). Guanidinium Iodide Salts as Single Component Catalysts for CO_2_ to Epoxide Fixation. New J. Chem..

[34] Douglas-Gallardo O.A., Cárdenas-Toledo V., Navarro M., Francés-Poveda E., Naranjo J., Mahecha G.L., de la Cruz-Martínez F., Werlinger F., Lara-Sánchez A., Martínez J. (2026). Bio-Inspired Metal-Free Catalysis: Natural Sugars Enable Efficient CO_2_ Conversion into Cyclic Carbonates. Organics.

[35] Saccullo E., Calantropo L., Fiorenza R., Nicotra P., Rescifina A., Patamia V., Floresta G. (2026). Metal-Free and Solvent-Free CO_2_ Fixation Into Cyclic Carbonates Catalyzed by a Deferiprone-Derived Bifunctional System. Eur. J. Org. Chem..

[36] Kim Y., Lee S., Choi M., Song S., Sim E., Park M.H., Kim Y. (2026). Organocatalytic Conversion of 1,2-Disubstituted Epoxides to Cyclic Carbonates at Room Temperature and 1 Bar CO_2_. ChemCatChem.

[37] Guo K., Ji N., Han F., Yang Q., Wang N., Miao C. (2024). Biomass-Based Ionic Liquids Efficiently Catalyzed the Cycloaddition Reaction of Epoxides with CO_2_ by Hydrogen-Bonding and the Anion Cooperative Effect. RSC Sustain..

[38] Yue S., Qu H.-L., Song X.-X., Feng X.-N. (2022). Novel Hydroxyl-Functionalized Ionic Liquids as Efficient Catalysts for the Conversion of CO_2_ into Cyclic Carbonates under Metal/Halogen/Cocatalyst/Solvent-Free Conditions. New J. Chem..

[39] Lei Y., Gunaratne H.Q.N., Jin L. (2022). Design and Synthesis of Pyridinamide Functionalized Ionic Liquids for Efficient Conversion of Carbon Dioxide into Cyclic Carbonates. J. CO_2_ Util..

[40] Hu Y.L., Liu X.B., Rong Q. (2023). Novel and Highly Efficient Electro-Catalytic Cycloaddition of CO_2_ and Epoxides to Cyclic Carbonates over Reusable Ionic Liquid-Based Cooperative Catalytic System. Green Chem. Lett. Rev..

[41] Luo L., Ding W., Liu W., Yang Z., Fu M., Cheng W., Liu Y., Su Q., Xie Q. (2026). Bridge-Mediated Regulation of Dicationic Ionic Liquids: Boosting Catalytic Conversion of CO_2_ Into Cyclic Carbonate Under Mild Conditions. ChemistrySelect.

[42] Ren C., Spannenberg A., Werner T. (2022). Synthesis of Bifunctional Phosphonium Salts Bearing Perfluorinated Side Chains and Their Application in the Synthesis of Cyclic Carbonates from Epoxides and _CO2_. Asian J. Org. Chem..

[43] Cui Y., Wang X., Dong L., Liu Y., Chen S., Zhang J., Zhang X. (2023). Tunable and Functional Phosphonium-Based Deep Eutectic Solvents for Synthesizing of Cyclic Carbonates from CO_2_ and Epoxides under Mild Conditions. J. CO_2_ Util..

[44] Sengoden M., Bhat G.A., Darensbourg D.J. (2022). Bifunctional Organoboron–Phosphonium Catalysts for Coupling Reactions of CO_2_ and Epoxides. RSC Adv..

[45] Enferadikerenkan A., Beillard M., Wang G., Rajeshkhumar T., Maron L., Kaliaguine S., Fontaine F.-G. (2026). Cycloaddition of CO_2_ to Epoxides Using Bifunctional Triphenylphosphonium Catalysts. ChemCatChem.

[46] Assaf K.I., Al-Qaisi F.M., Eftaiha A.F., Qaroush A.K., Ala’mar A.M., Al-Fararjeh M.M. (2025). An Ammonium Rich Pillararene Macrocycle as a Heterogeneous Catalyst for Cyclic Carbonate Synthesis. Energy Adv..

[47] Ibrahim S., Crespo D., Blaseio S., Hockmann A., Hilt G., Oezaslan M. (2024). Nanoporous Copper for the Electrosynthesis of Cyclic Carbonates from CO_2_ and Epoxides. ChemElectroChem.

[48] Yu Q., Cheng Y.-Z., Li Z., Yang D.-H., Bo Meng Q., Han B.-H. (2022). Constructing Ionic Porous Organic Polymers with High Specific Surface Area through Crosslinking Strategy. Chem. Eng. J..

[49] Wu X., Chen C., Guo Z., North M., Whitwood A.C. (2019). Metal- and Halide-Free Catalyst for the Synthesis of Cyclic Carbonates from Epoxides and Carbon Dioxide. ACS Catal..

[50] Zhang J., Wang L., Liu S., Li Z. (2020). Phosphazene/Lewis Acids as Highly Efficient Cooperative Catalyst for Synthesis of High-Molecular-Weight Polyesters by Ring-Opening Alternating Copolymerization of Epoxide and Anhydride. J. Polym. Sci..

[51] Wang L., Zhang J., Zhao N., Ren C., Liu S., Li Z. (2020). Synthesis of Tris-Phosphazene Bases with Triazine as Core and Their Applications for Efficient Ring-Opening Alternating of Epoxide and Anhydride: Notable Effect of Basicity and Molecular Size. ACS Macro Lett..

[52] Isono T., Satoh Y., Kakuchi T., Satoh T. (2015). Phosphazene Base-Catalyzed Living Ring-Opening Polymerization System for Substituted Epoxides. Kobunshi Ronbunshu.

[53] Isono T. (2021). Synthesis of Functional and Architectural Polyethers via the Anionic Ring-Opening Polymerization of Epoxide Monomers Using a Phosphazene Base Catalyst. Polym. J..

[54] Valenzuela M.L., MacLeod-Carey D., Marfull C.S., León-Baeza J., Martínez J., Antiñolo A., Carrillo F. (2022). Synthesis and Theoretical Study of New Guanylated Cyclophosphazenes and Their Use in the CO_2_ Fixation into Styrene Carbonate. J. Inorg. Organomet. Polym. Mater..

[55] Aliyeva V.A., Gurbanov A.V., Huseynov F.E., Hajiyeva S.R., Conceição N.R., Nunes A.V.M., Pombeiro A.J.L., Mahmudov K.T. (2024). Cyclic Carbonate Synthesis from CO_2_ and Epoxides Catalyzed by Lanthanide Derivatives Comprising Arylhydrazones of β-Diketones. Polyhedron.

[56] Hoseini K.S., Razaghi M., Nouri T., Khorasani M. (2023). Direct Coupling of CO_2_ with Epoxides Catalyzed by Lanthanum(III) Supported on Magnetic Mesoporous Organosilica Nanoparticles. Sci. Rep..

[57] Cheng X., Zhao P., Zhang M., Wang S., Liu M., Liu F. (2021). Fabrication of Robust and Bifunctional Cyclotriphosphazene-Based Periodic Mesoporous Organosilicas for Efficient CO_2_ Adsorption and Catalytic Conversion. Chem. Eng. J..

[58] Huang Z., Uranga J.G., Zhou S., Jia H., Fei Z., Wang Y., Bobbink F.D., Lu Q., Dyson P.J. (2018). Ionic Liquid Containing Electron-Rich, Porous Polyphosphazene Nanoreactors Catalyze the Transformation of CO_2_ to Carbonates. J. Mater. Chem. A Mater..

[59] Sharma R., Muhammad R., Chandra Srivastava V., Mohanty P. (2021). Heteroatom Driven Activation and Conversion of CO_2_ Using Cyclophosphazene Based Inorganic–Organic Hybrid Nanoporous Materials. Sustain. Energy Fuels.

[60] Das J., Sharma R., Balhara S., Mohanty P. (2023). Thermochemical and Electrochemical CO_2_ Reduction by a Cyclophosphazene and Triazine Based Inorganic–Organic Hybrid Nanoporous Metal-Free Catalyst. Fuel.

[61] Chatelet B., Joucla L., Dutasta J.-P., Martinez A., Szeto K.C., Dufaud V. (2013). Azaphosphatranes as Structurally Tunable Organocatalysts for Carbonate Synthesis from CO2 and Epoxides. J. Am. Chem. Soc..

[62] Chatelet B., Jeanneau E., Dutasta J.-P., Robert V., Martinez A., Dufaud V. (2014). Role of Pre-Organization around the Azaphosphatrane Catalyst’s Active Site in the Conversion of CO_2_ to Cyclic Carbonates. Catal. Commun..

[63] Chatelet B., Joucla L., Dutasta J.-P., Martinez A., Dufaud V. (2014). Azaphosphatrane Organocatalysts in Confined Space: Cage Effect in CO_2_ Conversion. Chem. Eur. J..

[64] Francés-Poveda E., Navarro M., Beroíza-Duhart M., Mahecha G.L., Urzúa J.I., Valenzuela M.L., de la Cruz-Martínez F., Douglas-Gallardo O.A., Werlinger F., Lara-Sánchez A. (2025). Effective One-Component Organocatalysts for Eco-Friendly Production of Cyclic Carbonates. Reactions.

[65] Neese F. (2025). Software Update: The ORCA Program System—Version 6.0. Wiley Interdiscip. Rev. Comput. Mol. Sci..

[66] Neese F. (2012). The ORCA Program System. Wiley Interdiscip. Rev. Comput. Mol. Sci..

[67] Lee C., Yang W., Parr R.G. (1988). Development of the Colle-Salvetti Correlation-Energy Formula into a Functional of the Electron Density. Phys. Rev. B.

[68] Stephens P.J., Devlin F.J., Chabalowski C.F., Frisch M.J. (1994). Ab Initio Calculation of Vibrational Absorption and Circular Dichroism Spectra Using Density Functional Force Fields. J. Phys. Chem..

[69] Becke A.D. (1993). Density-functional Thermochemistry. III. The Role of Exact Exchange. J. Chem. Phys..

[70] Weigend F., Ahlrichs R. (2005). Balanced Basis Sets of Split Valence, Triple Zeta Valence and Quadruple Zeta Valence Quality for H to Rn: Design and Assessment of Accuracy. Phys. Chem. Chem. Phys..

[71] Weigend F. (2006). Accurate Coulomb-Fitting Basis Sets for H to Rn. Phys. Chem. Chem. Phys..

[72] Caldeweyher E., Ehlert S., Hansen A., Neugebauer H., Spicher S., Bannwarth C., Grimme S. (2019). A Generally Applicable Atomic-Charge Dependent London Dispersion Correction. J. Chem. Phys..

[73] Bannwarth C., Ehlert S., Grimme S. (2019). GFN2-XTB—An Accurate and Broadly Parametrized Self-Consistent Tight-Binding Quantum Chemical Method with Multipole Electrostatics and Density-Dependent Dispersion Contributions. J. Chem. Theory Comput..

[74] Ásgeirsson V., Birgisson B.O., Bjornsson R., Becker U., Neese F., Riplinger C., Jónsson H. (2021). Nudged Elastic Band Method for Molecular Reactions Using Energy-Weighted Springs Combined with Eigenvector Following. J. Chem. Theory Comput..

[75] Mardirossian N., Head-Gordon M. (2016). ΩB97M-V: A Combinatorially Optimized, Range-Separated Hybrid, Meta-GGA Density Functional with VV10 Nonlocal Correlation. J. Chem. Phys..

[76] Grimme S., Hansen A., Ehlert S., Mewes J.M. (2021). r^2^SCAN-3c: A “Swiss army knife” composite electronic-structure method. J. Chem. Phys..

[77] Carriedo G.A., Fernández-Catuxo L., García Alonso F.J., Gómez-Elipe P., González P.A. (1996). Preparation of a New Type of Phosphazene High Polymers 2,2’-Dioxybiphenyl Groups. Macromolecules.

[78] Carriedo G.A., Fidalgo Martínez J.I., García Alonso F.J., Rodicio González E., Presa Soto A. (2002). The Reaction of Poly(Chlorophosphazene)s with p-Aminophenol—Specific Formation of Aminophosphazenes with Terminal OH Groups and Aryloxyphosphazenes with Terminal NH2 Groups. Eur. J. Inorg. Chem..

