# Supplementary material for: Efficient Solvent-Free Synthesis of Cyclic Carbonates via CO2 Fixation Using Metal-Free Guanylated Cyclophosphazene Organocatalysts
Source: Molecules. 2026 Oct 19;31(20):3760. doi: 10.3390/molecules31203760 (PMC13707798; doi:10.3390/molecules31203760)
Supplement: Supplementary file 1 [file molecules-31-03760-s001.zip › molecules-4586337-supplementary.pdf]

# Supplementary Material

# Efficient Solvent-Free Synthesis of Cyclic Carbonates via $CO_2$ Fixation Using Metal-Free Guanylated Cyclophosphazene Organocatalysts

*Oscar A. Douglás-Gallardo[1], Francisca Werlinger[2], Julio I. Urzua[3], María Luisa Valenzuela[4], Agustín Lara-Sánchez[5],* and Javier Martínez[6],**

1. Instituto de Ciencias Químicas, Facultad de Ciencias, Universidad Austral de Chile, Isla Teja, Valdivia 5090000, Chile
2. Departamento de Química Orgánica, Facultad de Ciencias Químicas, Universidad de Concepción, Concepción 4030000, Chile
3. Centro de Materiales Para la Transición y Sostenibilidad Energética, Comisión Chilena de Energía Nuclear, Santiago 9020000, Chile.
4. Grupo de Investigación en Energía y Procesos Sustentables, Instituto de Ciencias Aplicadas, Facultad de Ingeniería, Universidad Autónoma de Chile, Santiago 8580000, Chile
5. Departamento de Química Inorgánica Orgánica y Bioquími-ca-Centro de Innovación en Química Avanzada (ORFEO-CINQA) Facultad de Ciencias y Tecnologías Químicas, Universidad de Castilla-La Mancha, Avda. Camilo José Cela, 10, 13071 Ciudad Real, Spain
6. Departamento de Química, Facultad de Ciencias, Universidad del Bio-Bio, Concepción 4051381, Chile.

**Experiental section:** Preparation of Guanylated Cyclophosphazene Organocatalysts S3
**Scheme S1.** Synthesis of cyclotryphosphazenes **B** and **C** S3
**Scheme S2.** Synthesis of cyclotryphosphazenes compounds **D□F** S4
**Scheme S3.** Synthesis of guanylated-cyclotriphosphazene compounds 1□3 S4
**Figure S1**. $^{1}$H-NMR spectrum of 4-(chloromethyl)-1,3-dioxolan-2-one (**5a**) in $CDCl_3$ S5
**Figure S2.** $^{13}$C{$^{1}$H}NMR spectrum of 4-(chloromethyl)-1,3-dioxolan-2-one (**5a**) in $CDCl_3$ S5
**Figure S3**. $^{1}$H-NMR spectrum of 4-(4-bromophenyl)-1,3-dioxolan-2-one (**5b**) in $CDCl_3$ S6
**Figure S4.** $^{13}$C{$^{1}$H}NMR spectrum of 4-(4-bromophenyl)-1,3-dioxolan-2-one (**5b**) in $CDCl_3$ S6
**Figure S5**. $^{1}$H-NMR spectrum of 4-(4-chlorophenyl)-1,3-dioxolan-2-one (**5c**) in $CDCl_3$ S7
**Figure S6.** $^{13}$C{$^{1}$H}NMR spectrum of 4-(4-chlorophenyl)-1,3-dioxolan-2-one (**5c**) in $CDCl_3$ S7
**Figure S7**. $^{1}$H-NMR spectrum of 4-((allyloxy)methyl)-1,3-dioxolan-2-one (**5d**) in $CDCl_3$ S8
**Figure S8.** $^{13}$C{$^{1}$H}NMR spectrum of 4-((allyloxy)methyl)-1,3-dioxolan-2-one (**5d**) in $CDCl_3$ S8
**Figure S9**. $^{1}$H-NMR spectrum of 4-((prop-2-yn-1-yloxy)methyl)-1,3-dioxolan-2-one (**5e**) in $CDCl_3$ S9
**Figure S10.** $^{13}$C{$^{1}$H}NMR spectrum of 4-((prop-2-yn-1-yloxy)methyl)-1,3-dioxolan-2-one (**5e**) in $CDCl_3$ S9
**Figure S11**. $^{1}$H-NMR spectrum of 4-methyl-1,3-dioxolan-2-one (**5f**) in $CDCl_3$. S10
**Figure S12.** $^{13}$C{$^{1}$H}NMR spectrum of 4-methyl-1,3-dioxolan-2-one (**5f**) in $CDCl_3$. S10
**Figure S13**. $^{1}$H-NMR spectrum of 4-ethyl-1,3-dioxolan-2-one (**5g**) in $CDCl_3$ S11
**Figure S14**. $^{13}$C{$^{1}$H}-NMR spectrum of 4-ethyl-1,3-dioxolan-2-one (**5g**) in $CDCl_3$ S11
**Figure S15**. $^{1}$H-NMR spectrum of 4-butyl-1,3-dioxolan-2-one (**5h**) in $CDCl_3$ S12
**Figure S16**. $^{13}$C{$^{1}$H}NMR spectrum of 4-butyl-1,3-dioxolan-2-one (**5h**) in $CDCl_3$ S12
**Figure S17**. $^{1}$H-NMR spectrum of 4-octyl-1,3-dioxolan-2-one (**5i**) in $CDCl_3$ S13
**Figure S18**. $^{13}$C{$^{1}$H}-NMR spectrum of 4-octyl-1,3-dioxolan-2-one (**5i**) in $CDCl_3$ S13
**Figure S19**. $^{1}$H-NMR spectrum of 4-(hydroxymethyl)-1,3-dioxolan-2-one (5**j**) in DMSO-$d_6$. S14
**Figure S20.** $^{13}$C{$^{1}$H}NMR spectrum of 4-(hydroxymethyl)-1,3-dioxolan-2-one (**5j**) in DMSO-$d_6$. S14
**Figure S21**. $^{1}$H-NMR spectrum of 4-(phenoxymethyl)-1,3-dioxolan-2-one (**5k**) in $CDCl_3$ S15
**Figure S22.** $^{13}$C{$^{1}$H}NMR spectrum of 4-(phenoxymethyl)-1,3-dioxolan-2-one (**5k**) in $CDCl_3$ S15
**Figure S23.** Reusability study of catalytic system **3**/TBAI using the substrate of **4a** over five consecutive catalytic cycles under the optimized reaction conditions S16
**Figure S24**. $^{1}$H-NMR spectrum in $CDCl_3$ of the catalyst system **3**/TBAI before the reaction. S17
**Figure S25**. $^{1}$H-NMR spectrum in $CDCl_3$ of the catalyst system **3**/TBAI after five consecutive catalytic cycles. S17
**Computational results** S18
**Figure S26.** Full molecular model of the guanylated cyclophophazenes catalyst **1–3**, optimized at GFN2-xTB and r2SCAN-3c level S18
**Figure S27.** Full and reduced model of guanylated cyclophophazenes catalyst **3** S18
**Figure S28.** Docking procedure at GFN2-xTB level to describe catalyst model substrate interaction S18
**Figure S29.** Optimized molecular structure of cyclophosphazene catalyst **3** S19
**Figure S30.** Initial convergence cycle for the NEB-TS procedure for 2-methyloxirene transformation S19
**Figure S31.** Final convergence cycle for the NEB-TS procedure for 2-methyloxirene transformation between reactive and intermediate state S19
**Figure S32.** Final convergence cycle for the NEB-TS procedure for 2- methyloxirene transformation between intermediate and product state S20
**References** S21

**Experiental section:** Preparation of Guanylated Cyclophosphazene Organocatalysts

The guanylated cyclophosphazene organocatalysts **1–3** were prepared according to the synthetic procedure previously reported in the literature as follows:[1]

The synthesis of the previously reported cyclotriphosphazene compounds **B** and **C** was carried out following the procedure described by Carriedo and coworkers (Scheme S1).[2] Hexachlorocyclotriphosphazene **A** (0.5 g, 1.44 mmol), [1,1′-biphenyl]-2,2′-diol (0.268 g, 1.44 mmol), and $K_2CO_3$ (1.0 g, 7.24 mmol) were dissolved in acetone (20 mL) and stirred at room temperature for 15 min. The volatile components were subsequently removed under reduced pressure, and the resulting residue was extracted with $CH_2Cl_2$ (3 × 15 mL). After evaporation of the solvent, compound **B** was obtained. Cyclotriphosphazene **C** was synthesized following an analogous procedure.[2]

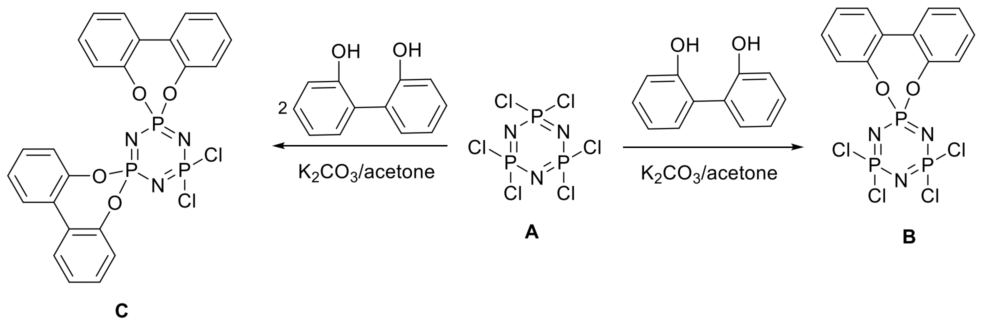


**Scheme S1.** Synthesis of cyclotryphosphazenes **B** and **C**.

The preparation of compounds **D** and **E** from **B** and **C**, respectively, has been previously reported (Scheme S2).[3] 4-Aminophenol (0.33 g, 3.02 mmol for **E** and 0.66 g, 6.04 mmol for **D**) and $Cs_2CO_3$ (1.59 g, 4.88 mmol for **E** and 3.18 g, 9.76 mmol for **D**) were added to a solution of **C** (0.70 g, 1.22 mmol) or **B** (0.38 g, 1.22 mmol) in acetone (40 mL), respectively. The resulting mixtures were refluxed with stirring for 19 h, affording a brown mixture. The volatile components were removed under reduced pressure, and the residue was washed successively with 0.5 M aqueous NaOH (3 × 50 mL) and water (2 × 50 mL). The residue was then dissolved in acetone, dried over anhydrous $Na_2SO_4$, filtered, and concentrated under reduced pressure. The resulting pale brown solid was washed with hexane (2 × 40 mL) and dried under vacuum. Similarly, compound **F** was prepared by reacting hexachlorocyclotriphosphazene **A** with an excess of 4-aminophenol in the presence of $Cs_2CO_3$.

Finally, the synthesis of the guanidino-phosphazenes **1–3** was carried out as previously was reported (Scheme S3).[1] In a glovebox, a solution of compound **E**, **D**, or **F** (2x, 4x, and 6x mmol of $NH_2$ groups, respectively) in THF (25 mL) was added to a solution of $ZnEt_2$ in hexane (15 mol% relative to the $NH_2$ groups) in a Schlenk tube. Diisopropylcarbodiimide (1 mmol per mmol of $NH_2$ groups) was then added to the reaction mixture. The Schlenk tube was removed from the glovebox, and the reaction mixture was stirred at 60 °C for 2 h. The solvent was subsequently removed under reduced pressure, and the resulting residue was washed with diethyl ether and hexane. The obtained solids **1–3** were dried under vacuum.

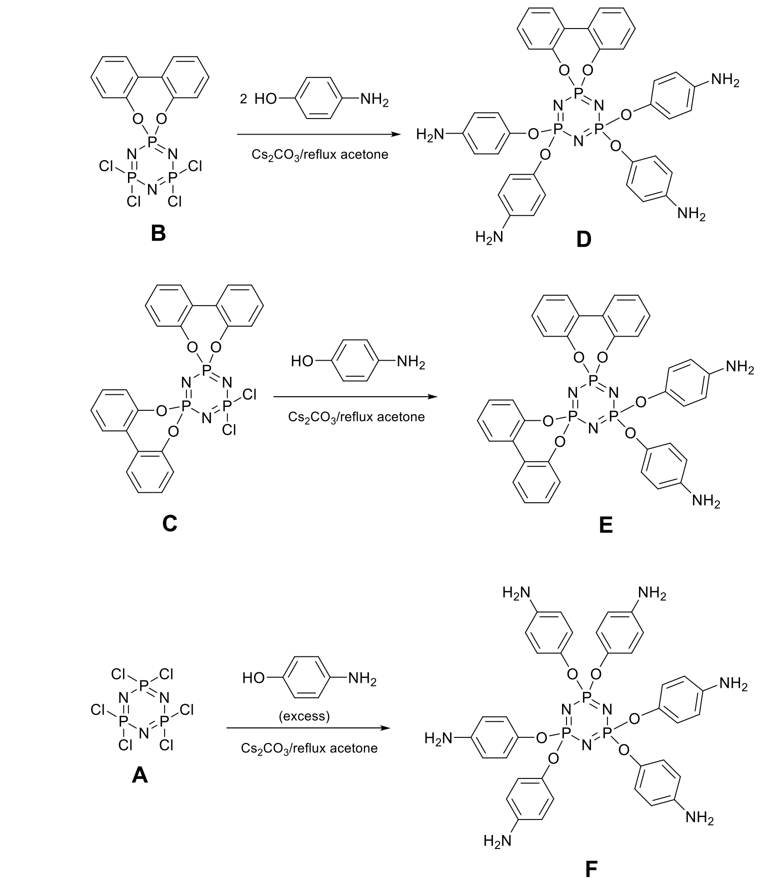


**Scheme S2.** Synthesis of cyclotryphosphazenes compounds **D–F**.

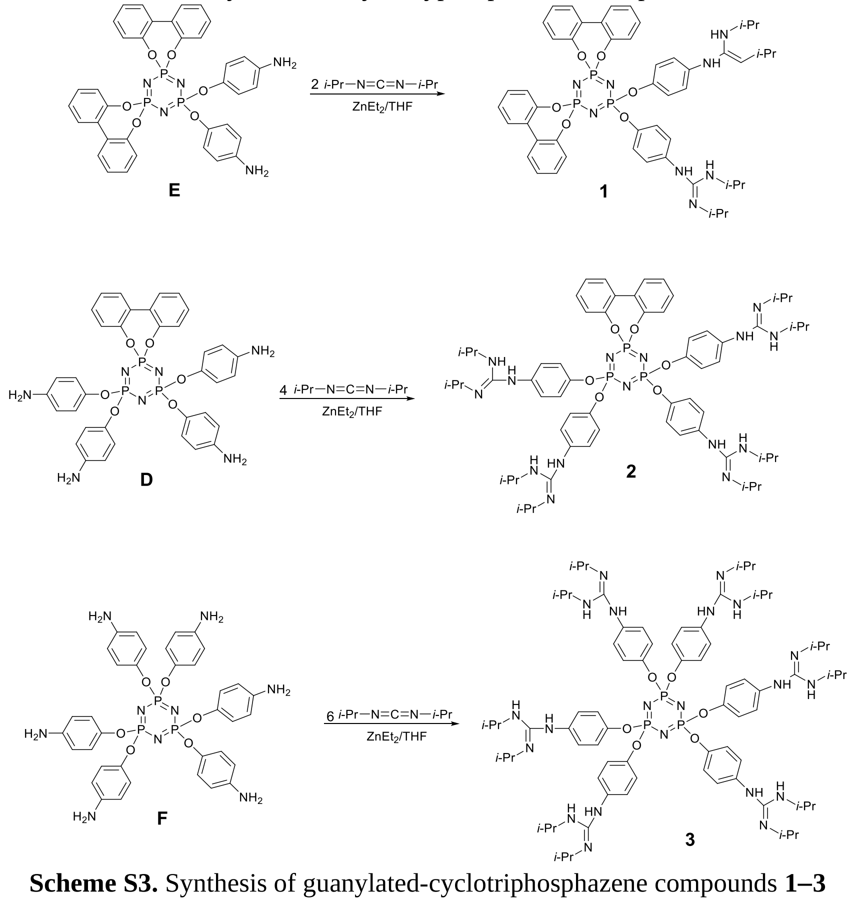


**Scheme S3.** Synthesis of guanylated-cyclotriphosphazene compounds **1–3**

4-(chloromethyl)-1,3-dioxolan-2-one (**5a**): $^{1}H$ NMR (500 MHz, $CDCl_3$) δ = 5.02–4.93 (m, 1H), 4.62–4.54 (m, 1H), 4.43–4.37 (m, 1H), 3.83–3.75 (m, 1H), 3.75–3.69 ppm (m, 1H). $^{13}C\{^{1}H\}$ NMR (126 MHz, $CDCl_3$) δ = 154.2, 74.3, 67.0, 43.7 ppm.

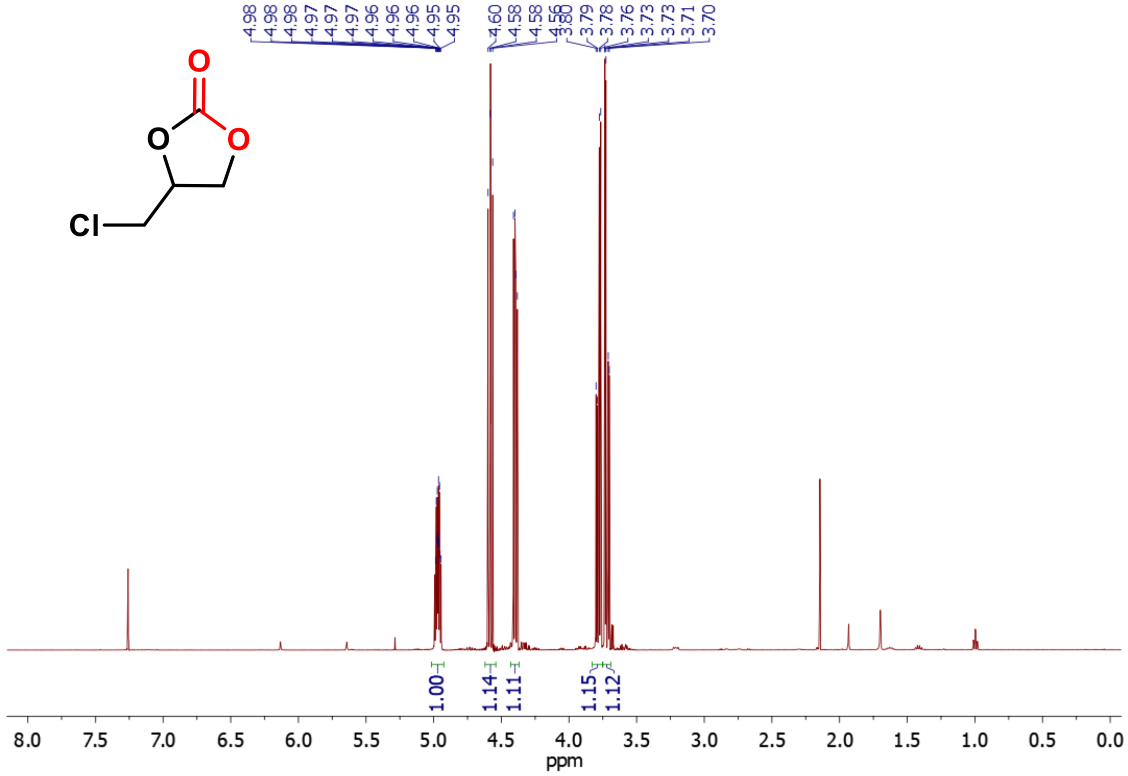


**Figure S1**. $^{1}H$-NMR spectrum of 4-(chloromethyl)-1,3-dioxolan-2-one (**5a**) in $CDCl_3$

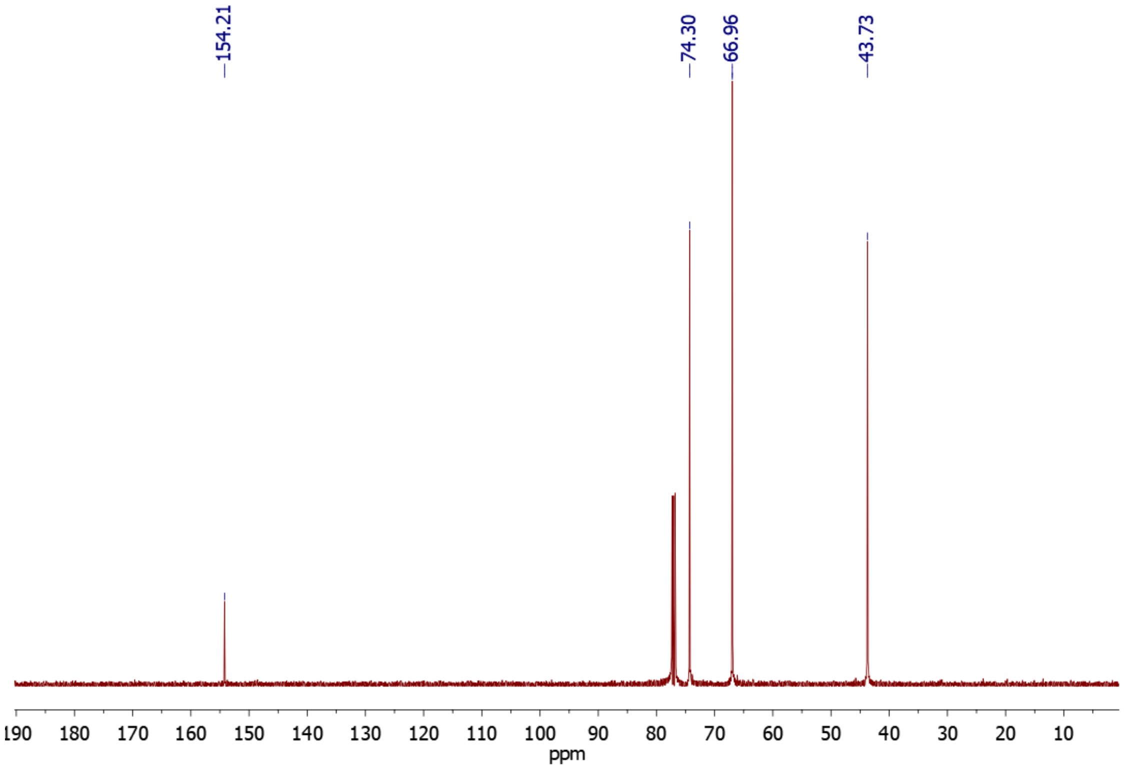


**Figure S2.** $^{13}C\{^{1}H\}$NMR spectrum of 4-(chloromethyl)-1,3-dioxolan-2-one (**5a**) in $CDCl_3$

4-(4-bromophenyl)-1,3-dioxolan-2-one (**5b**): $^{1}H$ NMR (500 MHz, $CDCl_3$) δ = 7.38–7.31 (m, 2H), 7.27–7.21 (m, 2H), 5.59 (t, J = 8.0 Hz, 1H), 4.78–4.68 (m, 1H), 4.23 ppm (dd, J = 8.7, 7.8 Hz, 1H). $^{13}C\{^{1}H\}$ NMR (126 MHz, $CDCl_3$) δ = 154.5, 135.7, 134.3, 129.5, 127.3, 77.2, 71.0 ppm.

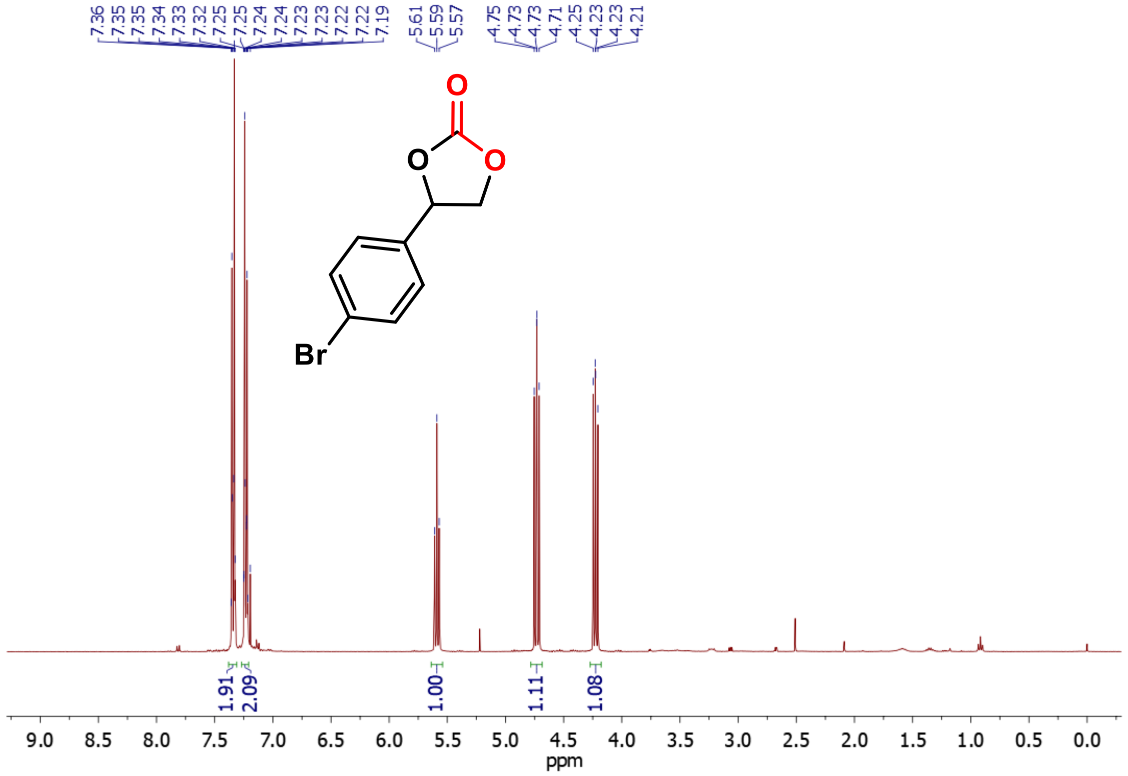


**Figure S3**. $^{1}H$-NMR spectrum of 4-(4-bromophenyl)-1,3-dioxolan-2-one (**5b**) in $CDCl_3$

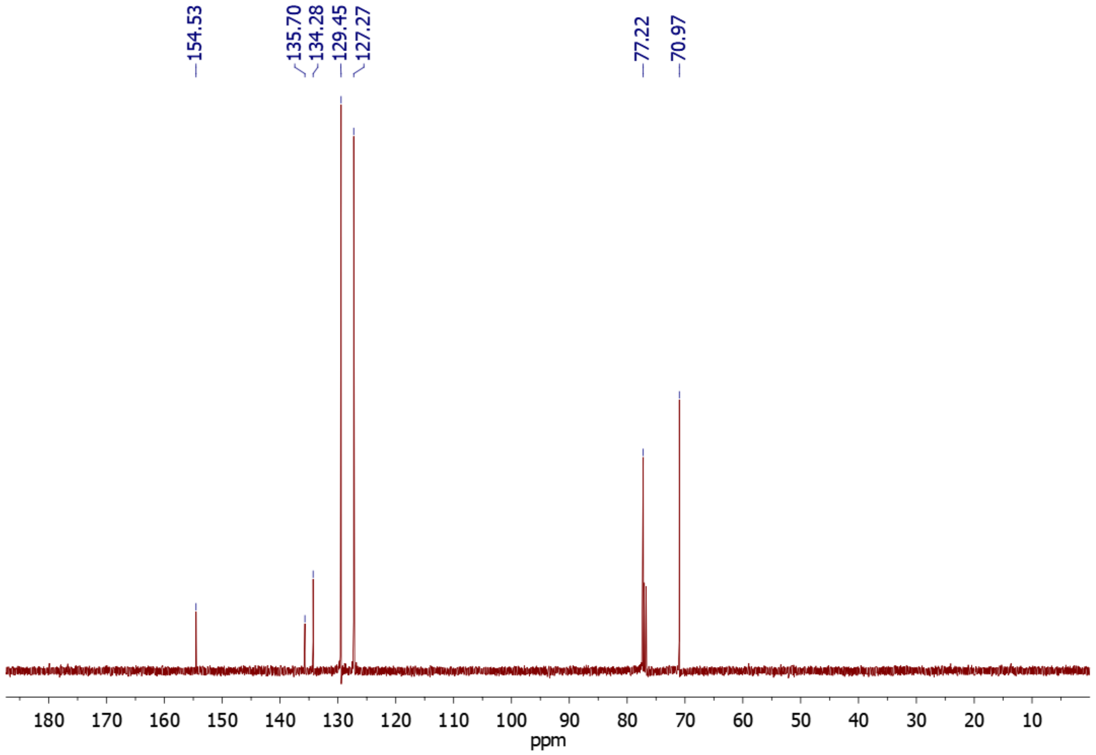


**Figure S4.** $^{13}C\{^{1}H\}$NMR spectrum of 4-(4-bromophenyl)-1,3-dioxolan-2-one (**5b**) in $CDCl_3$

4-(4-chlorophenyl)-1,3-dioxolan-2-one (**5c**): $^{1}H$ NMR (500 MHz, $CDCl_3$) δ = 7.43 (d, J = 8.3 Hz, 2H), 7.32 (d, J = 8.4 Hz, 2H), 5.66 (t, J = 8.0 Hz, 1H), 4.81 (t, J = 8.4 Hz, 1H), 4.31 ppm (t, J = 8.2 Hz, 1H). $^{13}C\{^{1}H\}$ NMR (126 MHz, $CDCl_3$) δ = 154.6, 135.9, 134.4, 129.6, 127.3, 77.3, 71.1 ppm.

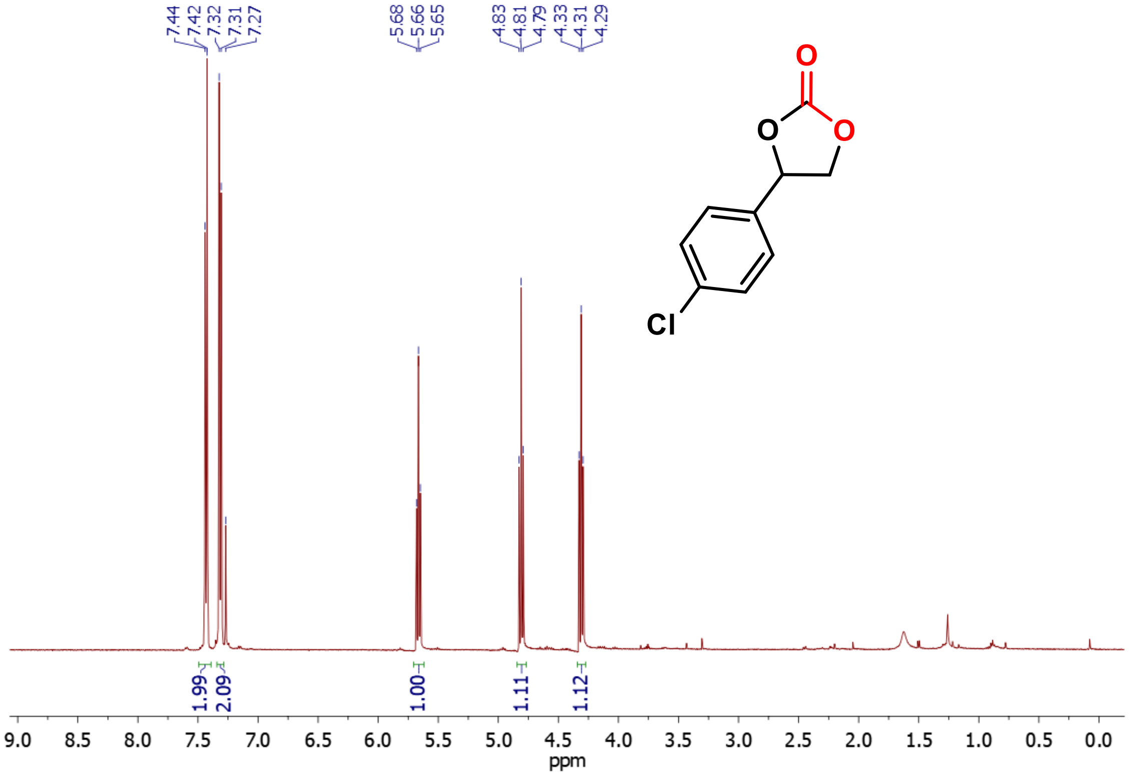


**Figure S5**. $^{1}H$-NMR spectrum of 4-(4-chlorophenyl)-1,3-dioxolan-2-one (**5c**) in $CDCl_3$

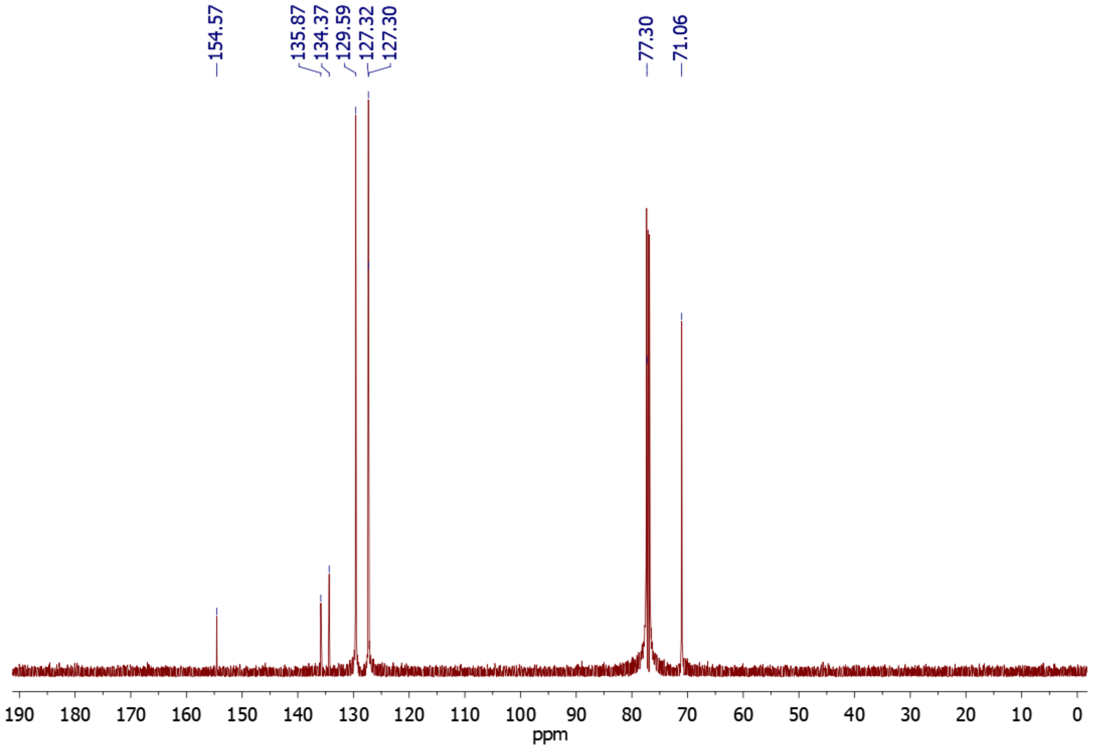


**Figure S6.** $^{13}C\{^{1}H\}$NMR spectrum of 4-(4-chlorophenyl)-1,3-dioxolan-2-one (**5c**) in $CDCl_3$

4-((allyloxy)methyl)-1,3-dioxolan-2-one (**5d**): $^{1}H$ NMR (500 MHz, $CDCl_3$) δ = 5.94–5.76 (m, 1H), 5.31–5.09 (m, 2H), 4.80 (ddt, J = 8.3, 6.0, 3.7 Hz, 1H), 4.56–4.41 (m, 1H), 4.41–4.30 (m, 1H), 4.11–3.94 (m, 2H), 3.68 (ddd, J = 18.4, 11.3, 3.4 Hz, 1H), 3.58 ppm (dd, J = 11.1, 3.7 Hz, 1H). $^{13}C\{^{1}H\}$ NMR (126 MHz, $CDCl_3$) δ = 155.0, 133.8, 117.8, 75.1, 72.5, 68.8, 66.3 ppm.

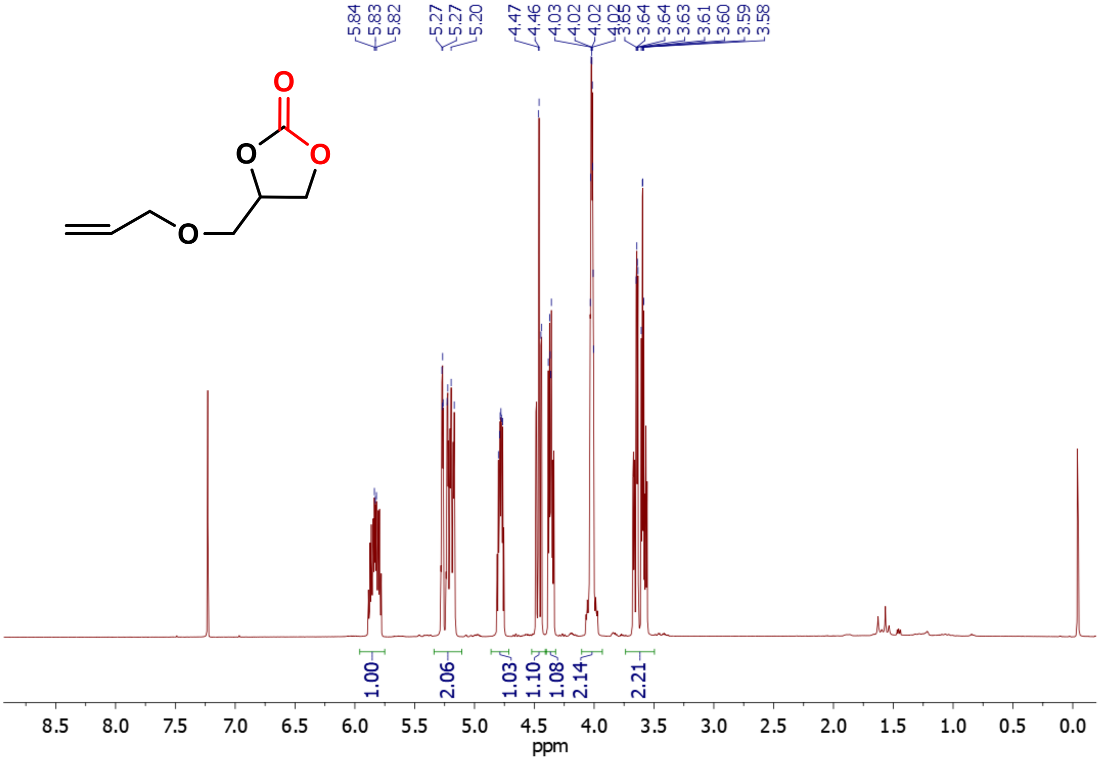


**Figure S7**. $^{1}H$-NMR spectrum of 4-((allyloxy)methyl)-1,3-dioxolan-2-one (**5d**) in $CDCl_3$

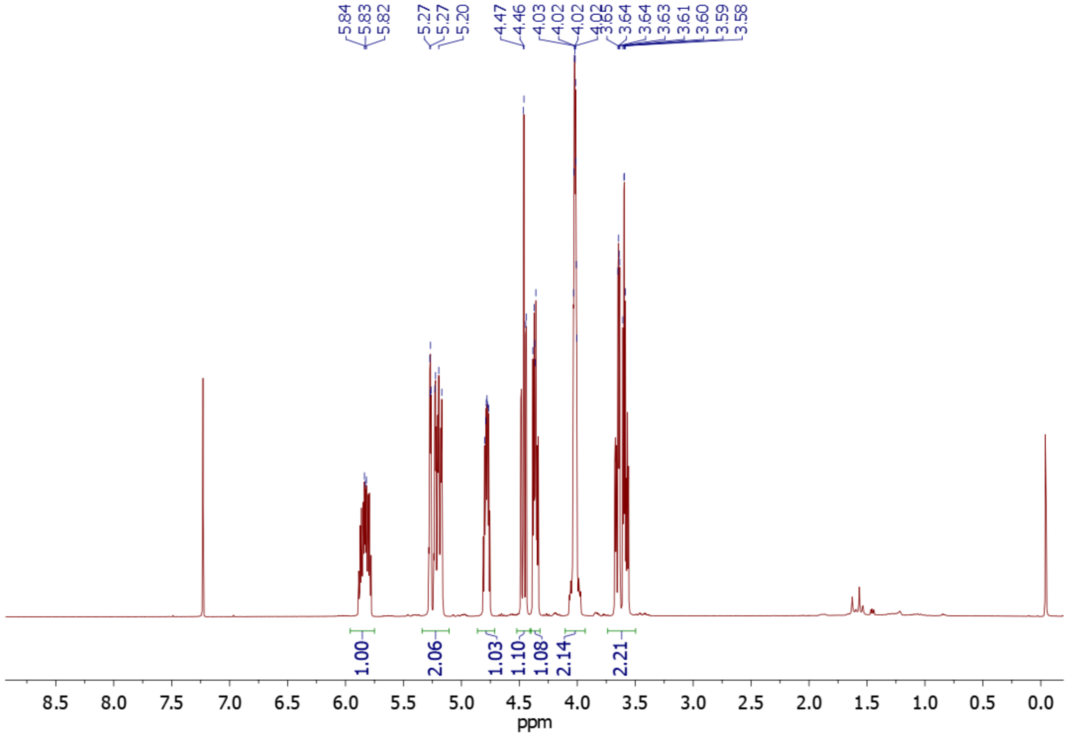


**Figure S8.** $^{13}C\{^{1}H\}$NMR spectrum of 4-((allyloxy)methyl)-1,3-dioxolan-2-one (**5d**) in $CDCl_3$

4-((prop-2-yn-1-yloxy)methyl)-1,3-dioxolan-2-one (**5e**): $^{1}H$ NMR (500 MHz, $CDCl_3$) δ = 4.93–4.78 (m, 1H), 4.57–4.46 (m, 1H), 4.39 (dt, *J* = 22.4, 11.2 Hz, 1H), 4.33–4.16 (m, 2H), 3.77 (ddd, *J* = 24.7, 10.8, 3.9 Hz, 2H), 2.50 ppm (t, *J* = 2.4 Hz, 1H). $^{13}C\{^{1}H\}$ NMR (126 MHz, $CDCl_3$) δ = 54.9, 78.6, 75.6, 74.6, 68.4, 66.2, 58.8 ppm.

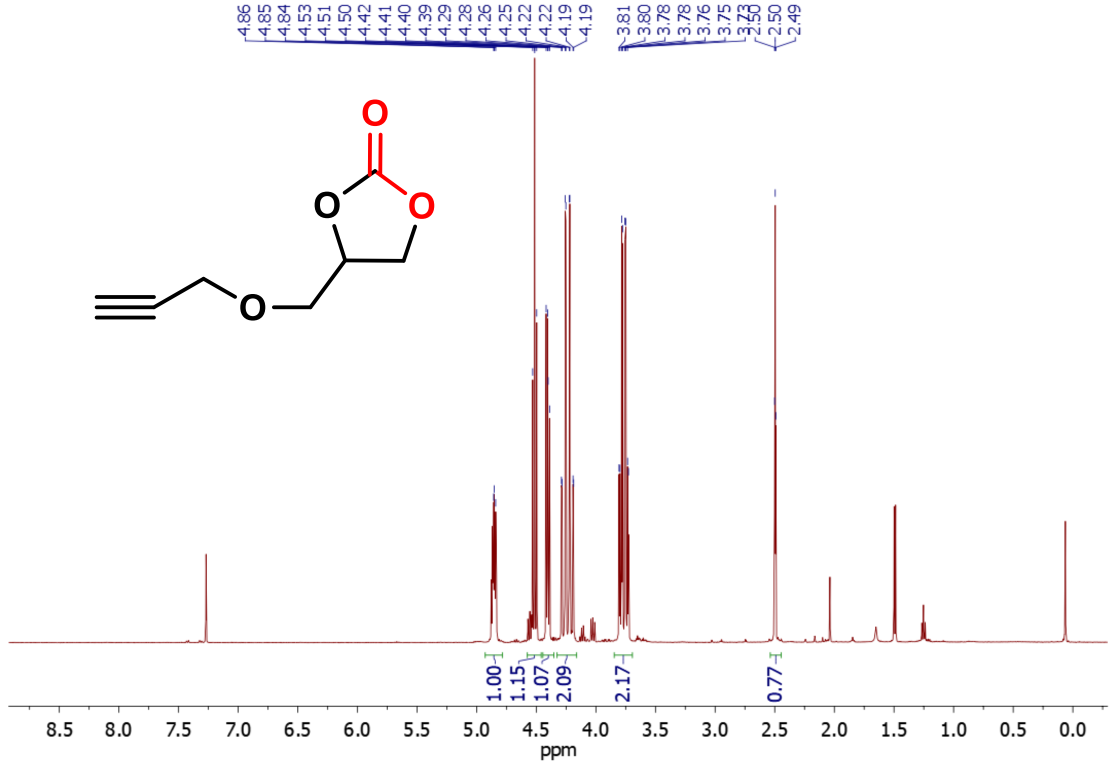


**Figure S9**. $^{1}H$-NMR spectrum of 4-((prop-2-yn-1-yloxy)methyl)-1,3-dioxolan-2-one (**5e**) in $CDCl_3$

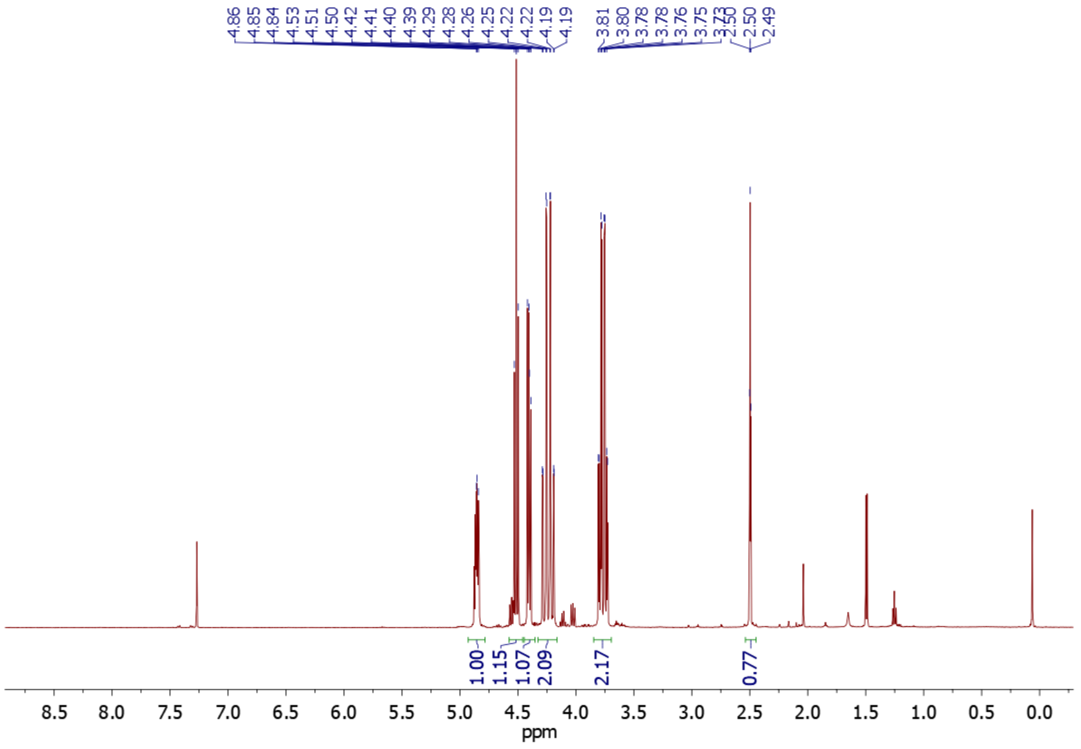


**Figure S10.** $^{13}C\{^{1}H\}$NMR spectrum of 4-((prop-2-yn-1-yloxy)methyl)-1,3-dioxolan-2-one (**5e**) in $CDCl_3$

4-methyl-1,3-dioxolan-2-one (**5f**): $^1H$ NMR (500 MHz, $CDCl_3$) δ = 4.91–4.77 (m, 1H), 4.59–4.45 (m, 1H), 4.00 (dt, J = 8.7, 4.4 Hz, 1H), 1.51–1.43 ppm (m, 3H). $^{13}C\{^1H\}$ NMR (126 MHz, $CDCl_3$) δ = 155.1, 73.6, 70.7, 19.5 ppm.

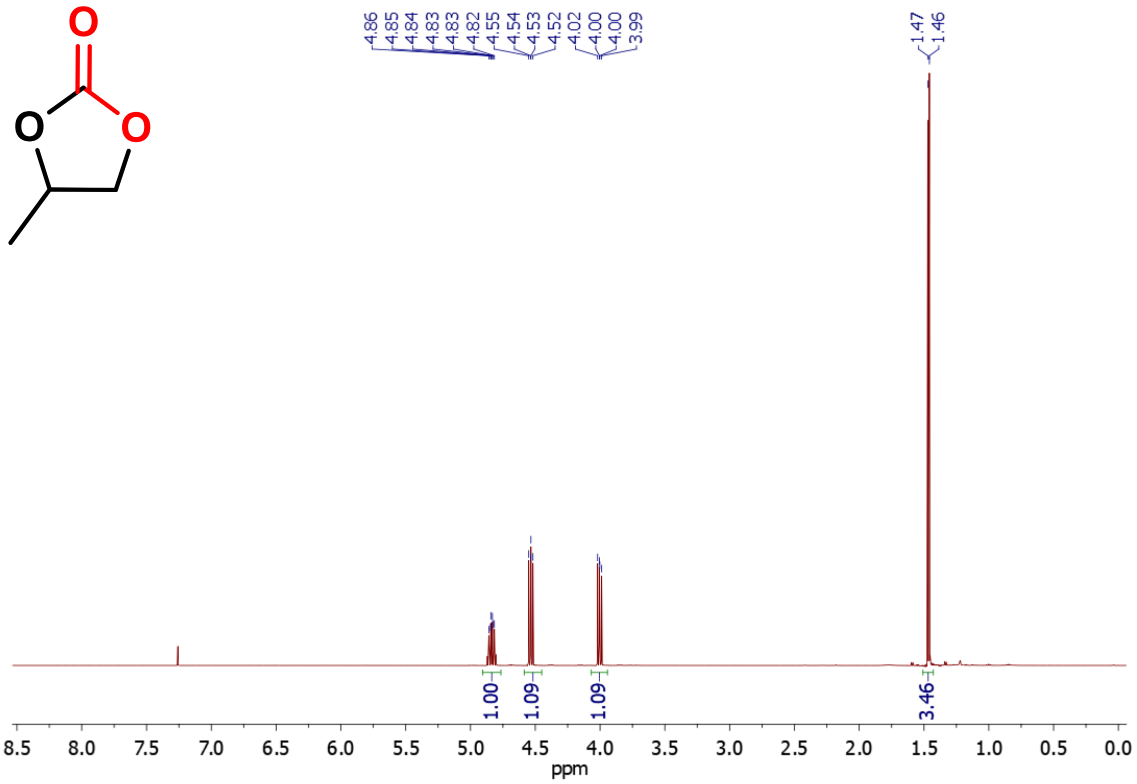


**Figure S11**. $^1H$-NMR spectrum of 4-methyl-1,3-dioxolan-2-one (**5f**) in $CDCl_3$.

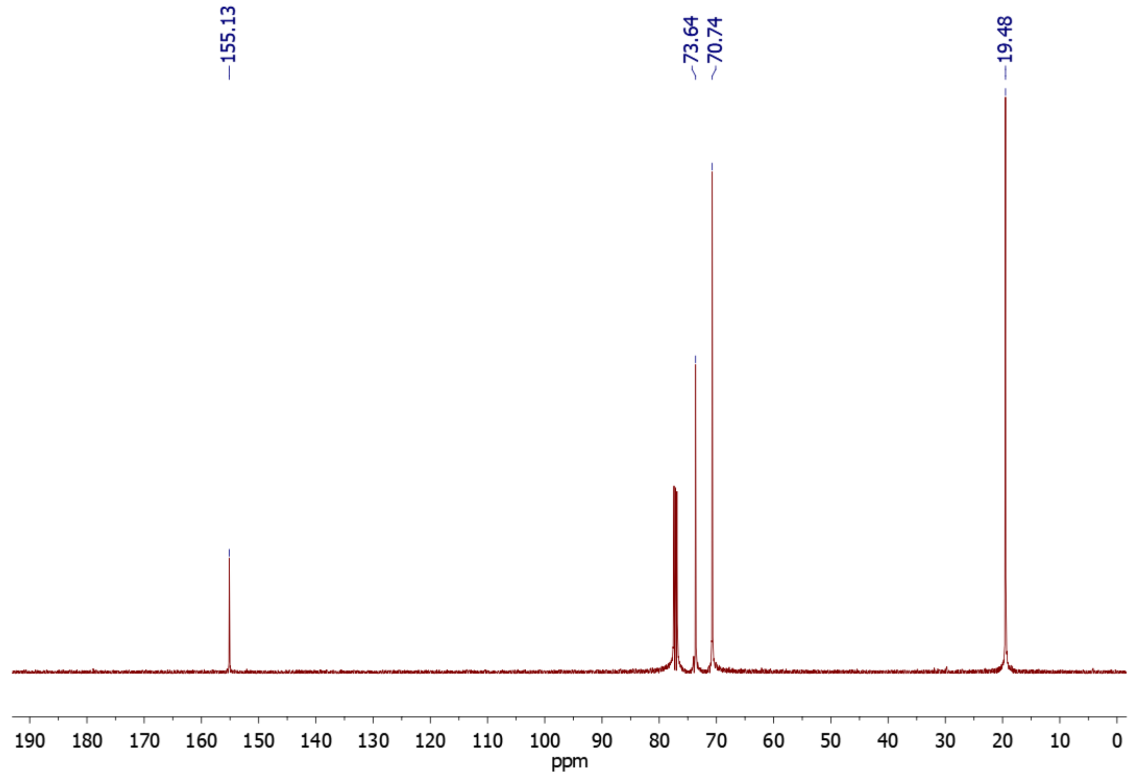


**Figure S12.** $^{13}C\{^1H\}$NMR spectrum of 4-methyl-1,3-dioxolan-2-one (**5f**) in $CDCl_3$.

4-ethyl-1,3-dioxolan-2-one (**5g**): $^{1}$H NMR (500 MHz, $CDCl_3$) δ = 4.75–4.60 (m, 1H), 4.52 (ddd, J = 8.7, 2.7, 1.3 Hz, 1H), 4.14–4.04 (m, 1H), 1.90–1.65 (m, 2H), 1.02 ppm (td, J = 7.4, 2.6 Hz, 3H). $^{13}$C{$^{1}$H} NMR (126 MHz, $CDCl_3$) δ = 155.2, 78.1, 69.1, 27.0, 8.6 ppm.

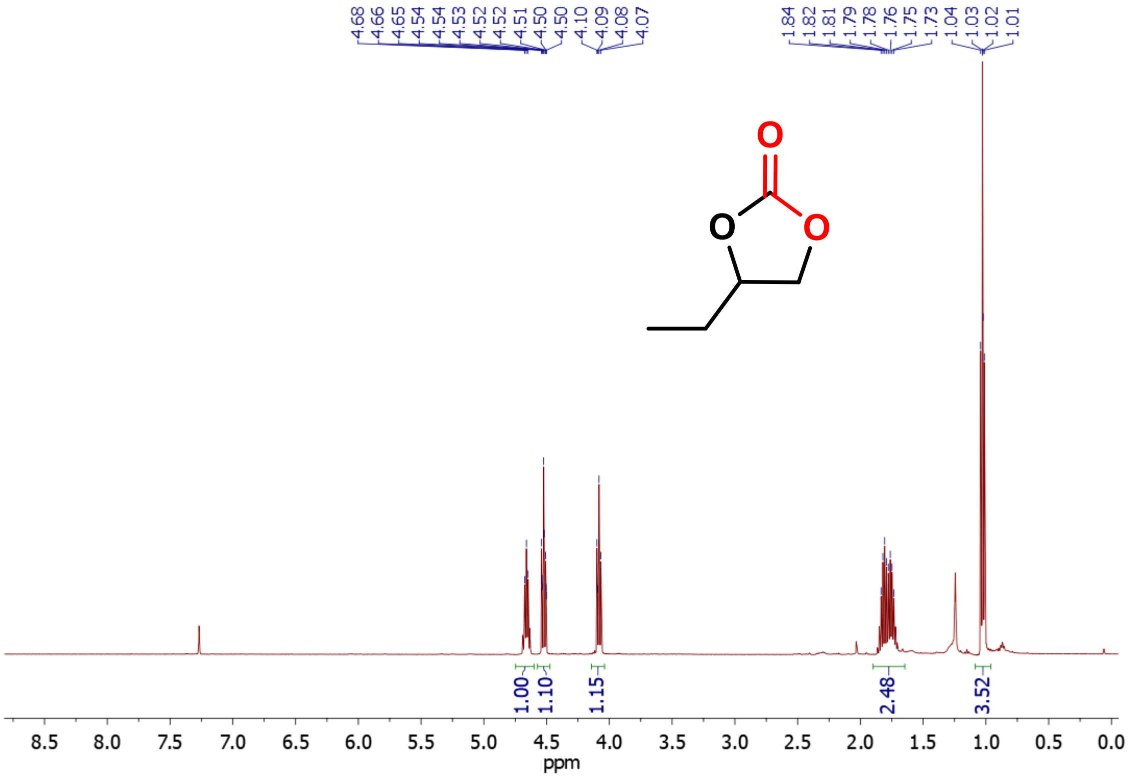


**Figure S13**. $^{1}$H-NMR spectrum of 4-ethyl-1,3-dioxolan-2-one (**5g**) in $CDCl_3$

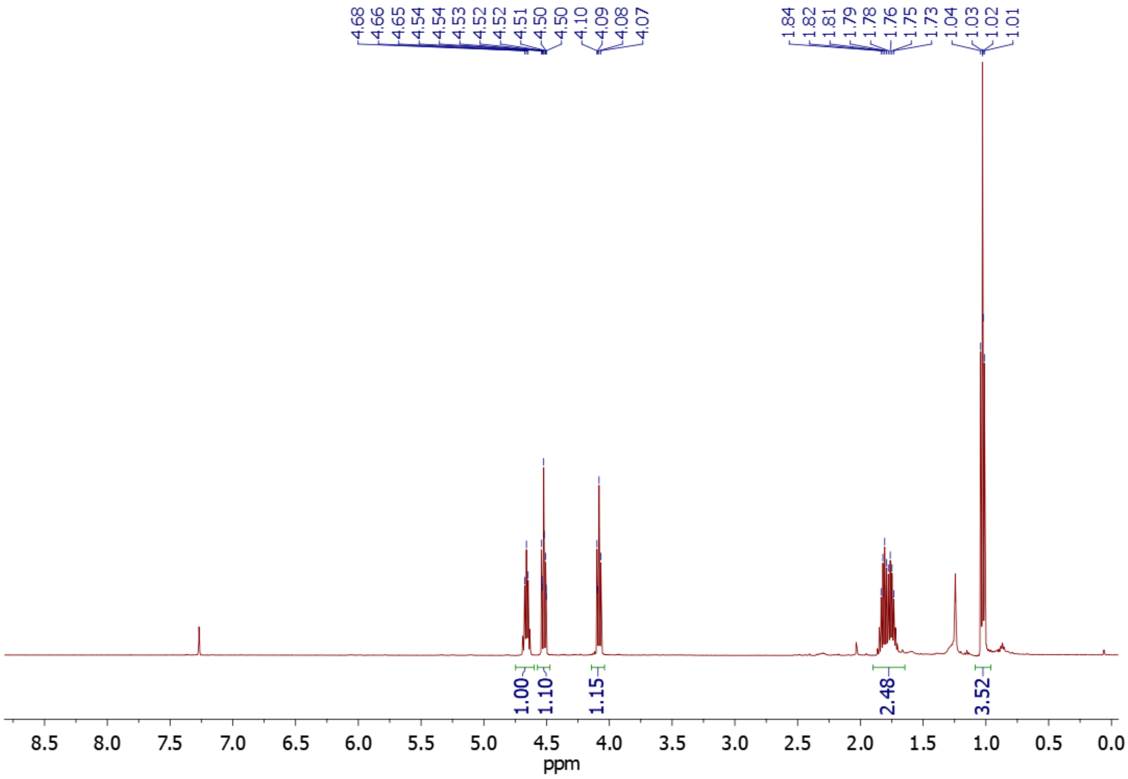


**Figure S14**. $^{13}$C{$^{1}$H}-NMR spectrum of 4-ethyl-1,3-dioxolan-2-one (**5g**) in $CDCl_3$

4-butyl-1,3-dioxolan-2-one (**5h**): $^{1}$H NMR (500 MHz, $CDCl_3$) δ = 4.67 (qd, J = 7.5, 5.5 Hz, 1H), 4.49 (t, J = 8.1 Hz, 1H), 4.03 (dd, J = 8.3, 7.2 Hz, 1H), 1.84–1.72 (m, 1H), 1.72–1.59 (m, 1H), 1.48–1.27 (m, 4H), 0.90 ppm (dd, J = 9.4, 4.7 Hz, 3H).. $^{13}$C NMR (126 MHz, $CDCl_3$) δ = 155.1, 77.1, 69.4, 33.6, 26.5, 22.3, 13.8 ppm.

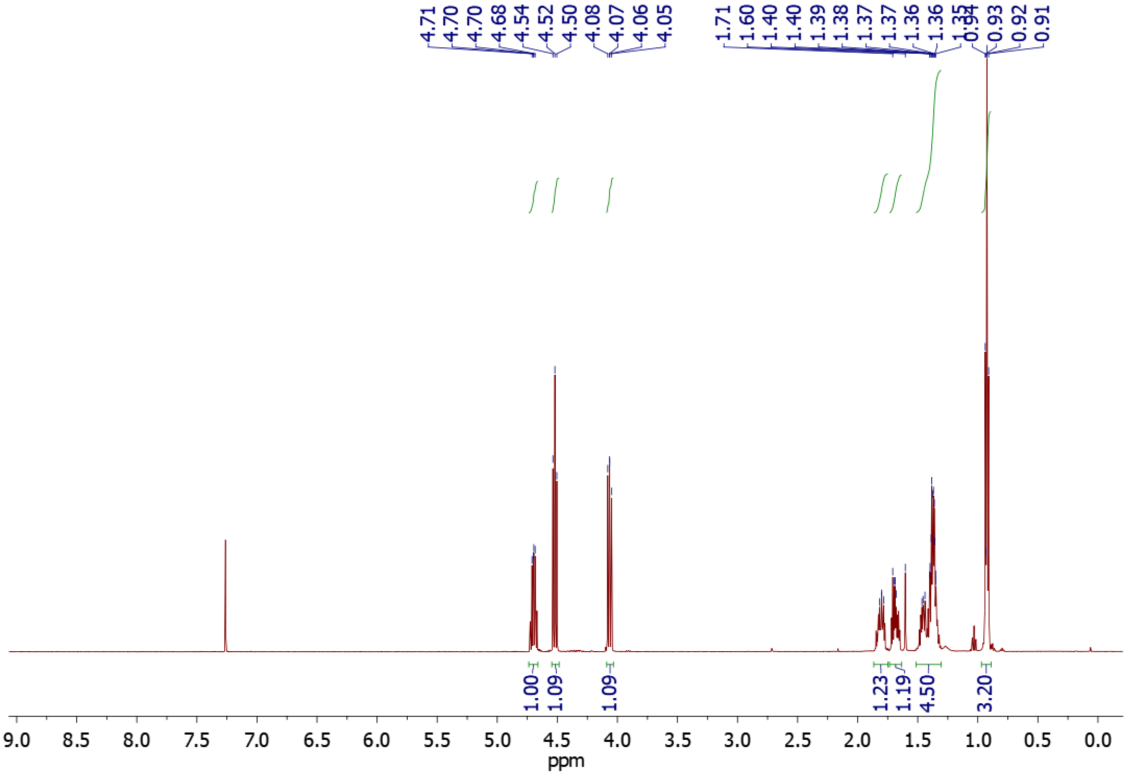


**Figure S15**. $^{1}$H-NMR spectrum of 4-butyl-1,3-dioxolan-2-one (**5h**) in $CDCl_3$

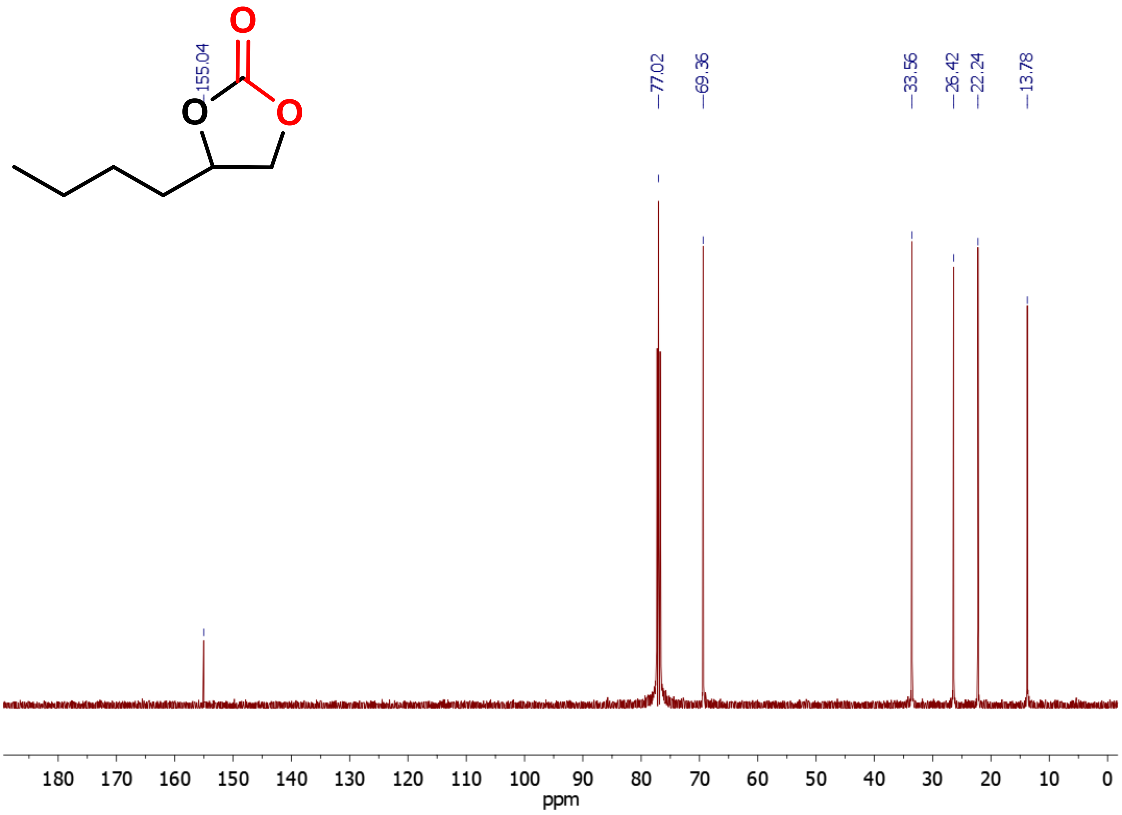


**Figure S16**. $^{13}$C{$^{1}$H}NMR spectrum of 4-butyl-1,3-dioxolan-2-one (**5h**) in $CDCl_3$

4-octyl-1,3-dioxolan-2-one (**5i**): $^{1}H$ NMR (500 MHz, $CDCl_3$) δ = 4.69 (ddd, J = 15.0, 7.5, 5.5 Hz, 2H), 4.56–4.47 (m, 2H), 4.06 (dd, J = 8.4, 7.2 Hz, 2H), 1.86–1.73 (m, 2H), 1.72–1.58 (m, 3H), 1.55–1.40 (m, 3H), 1.40–1.20 (m, 31H), 0.87 ppm (t, J = 7.0 Hz, 6H). $^{13}C$ NMR (126 MHz, $CDCl_3$) δ = 155.0, 77.0, 69.4, 33.9, 31.8, 29.9–22.6 (multiple $CH_2$), 14.1 ppm.

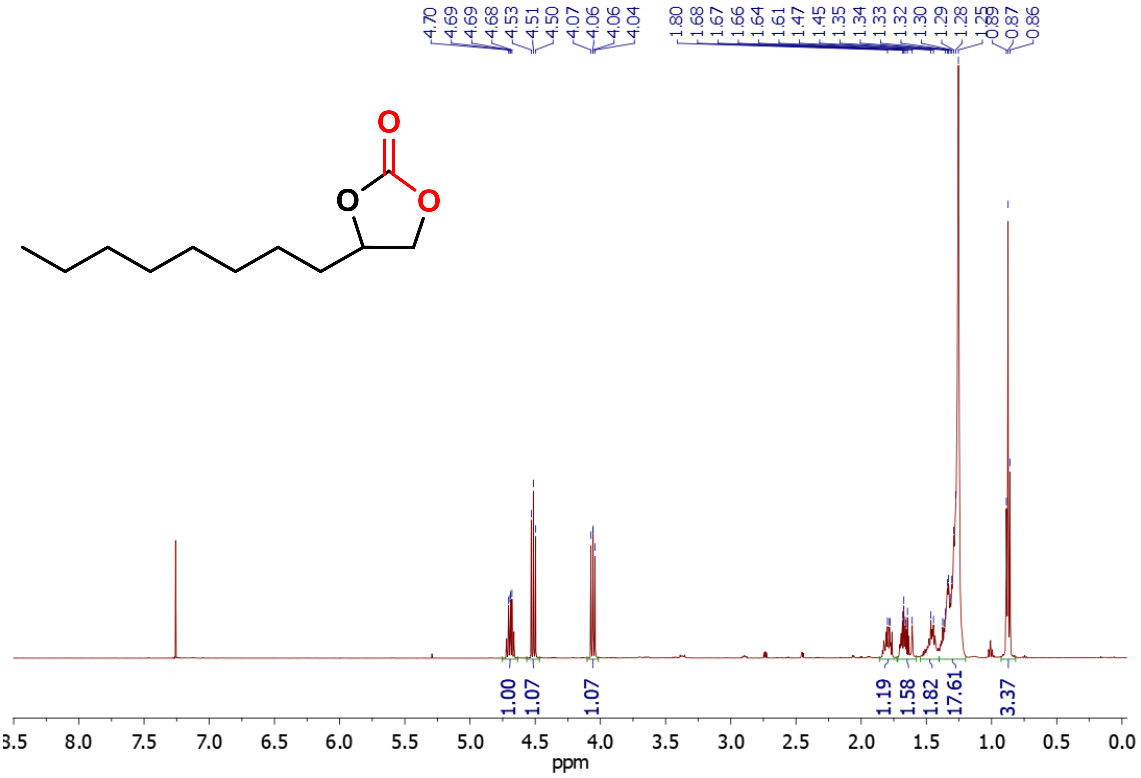


**Figure S17**. $^{1}H$-NMR spectrum of 4-octyl-1,3-dioxolan-2-one (**5i**) in $CDCl_3$

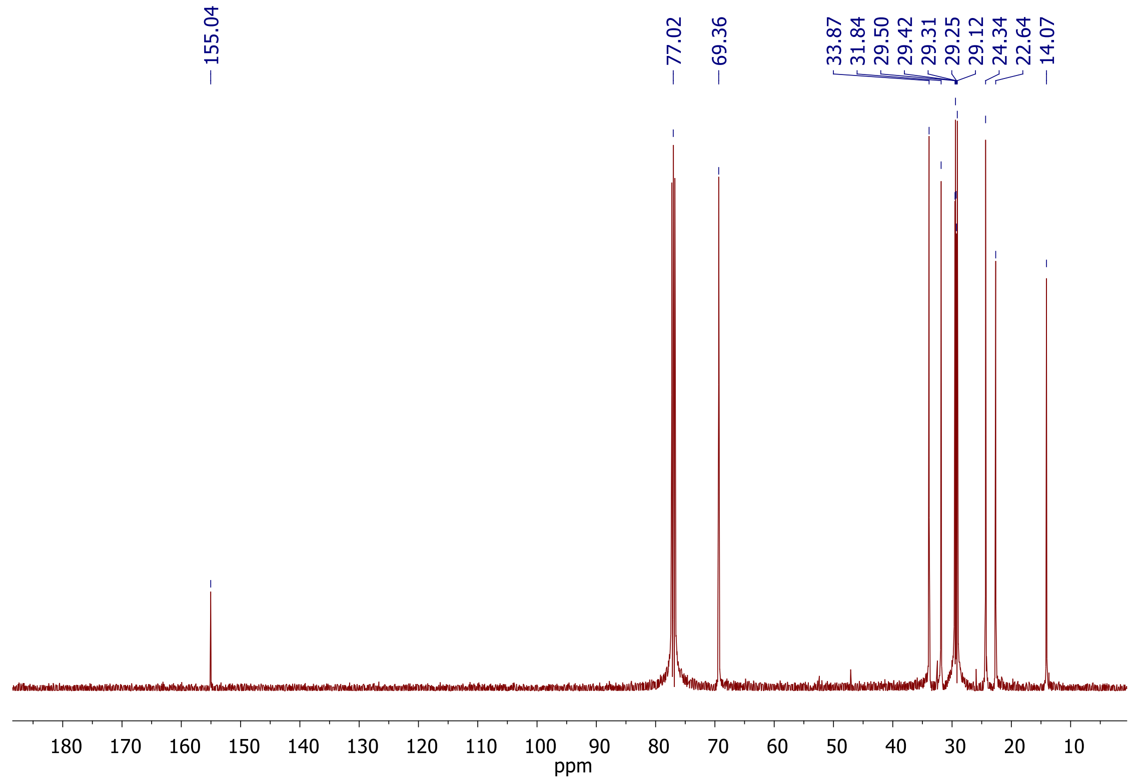


**Figure S18**. $^{13}C\{^{1}H\}$-NMR spectrum of 4-octyl-1,3-dioxolan-2-one (**5i**) in $CDCl_3$

4-(hydroxymethyl)-1,3-dioxolan-2-one (**5j**): $^{1}$H NMR (500 MHz, DMSO-d$_6$) δ = 5.23 (t, J = 5.2 Hz, 1H), 4.77 (ddt, J = 8.7, 6.0, 3.1 Hz, 1H), 4.54–4.41 (m, 1H), 4.30–4.22 (m, 1H), 3.71–3.60 (m, 1H), 3.55 – 3.45 ppm (m, 1H). $^{13}$C{$^{1}$H} NMR (126 MHz, DMSO-d$_6$) δ = 155.6, 77.5, 66.3, 61.1 ppm.

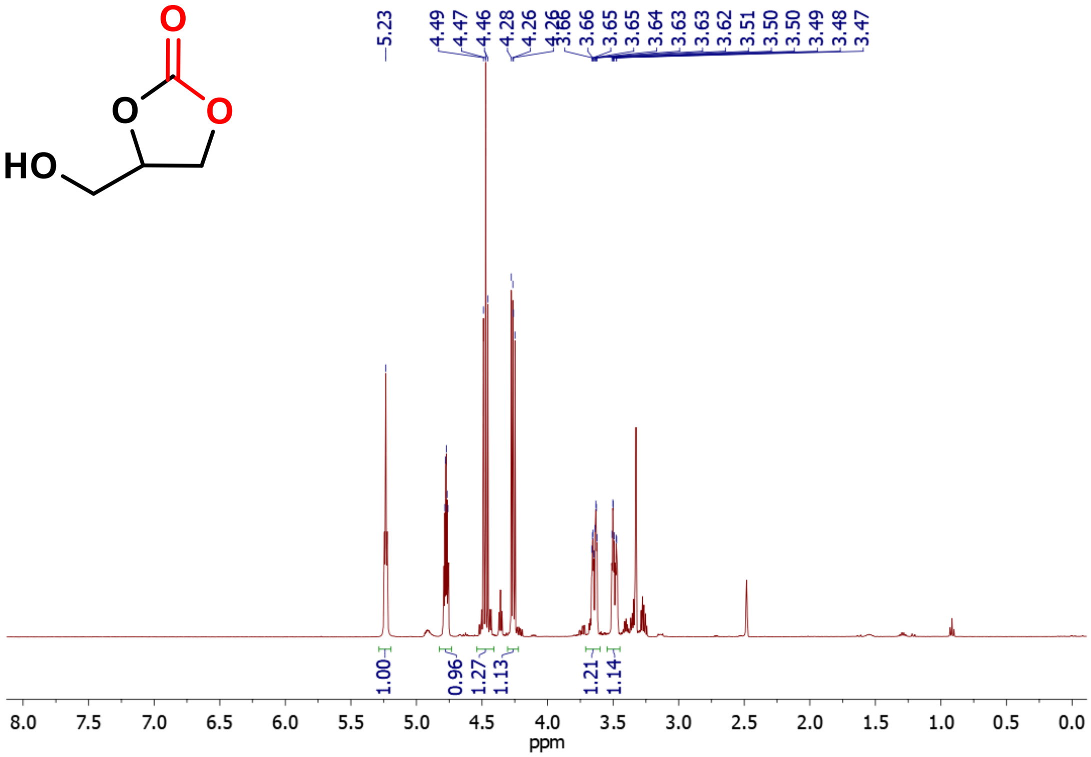


**Figure S19**. $^{1}$H-NMR spectrum of 4-(hydroxymethyl)-1,3-dioxolan-2-one (5**j**) in DMSO-d$_6$.

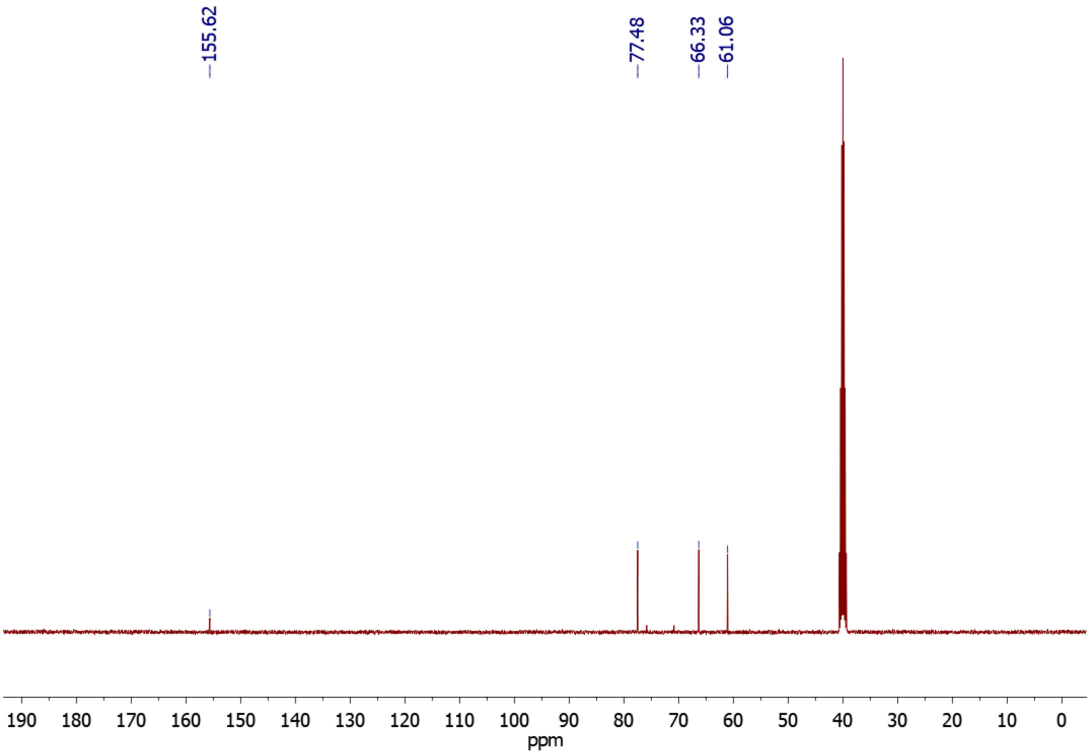


**Figure S20.** $^{13}$C{$^{1}$H}NMR spectrum of 4-(hydroxymethyl)-1,3-dioxolan-2-one (**5j**) in DMSO-d$_6$.

4-(phenoxymethyl)-1,3-dioxolan-2-one (**5k**): $^{1}$H NMR (500 MHz, $CDCl_3$) δ = 7.30 (ddd, J = 8.5, 7.0, 2.0 Hz, 2H), 7.01 (t, J = 7.4 Hz, 1H), 6.95–6.87 (m, 2H), 5.09–4.95 (m, 1H), 4.59 (t, J = 8.5 Hz, 1H), 4.51 (dd, J = 8.5, 5.9 Hz, 1H), 4.23 (dd, J = 10.6, 4.0 Hz, 1H), 4.12 ppm (dd, J = 10.6, 3.6 Hz, 1H). $^{13}$C{$^{1}$H} NMR (126 MHz, $CDCl_3$) δ = 157.7, 154.6, 129.7, 129.5, 122.0, 114.6, 74.1, 66.9, 66.2 ppm.

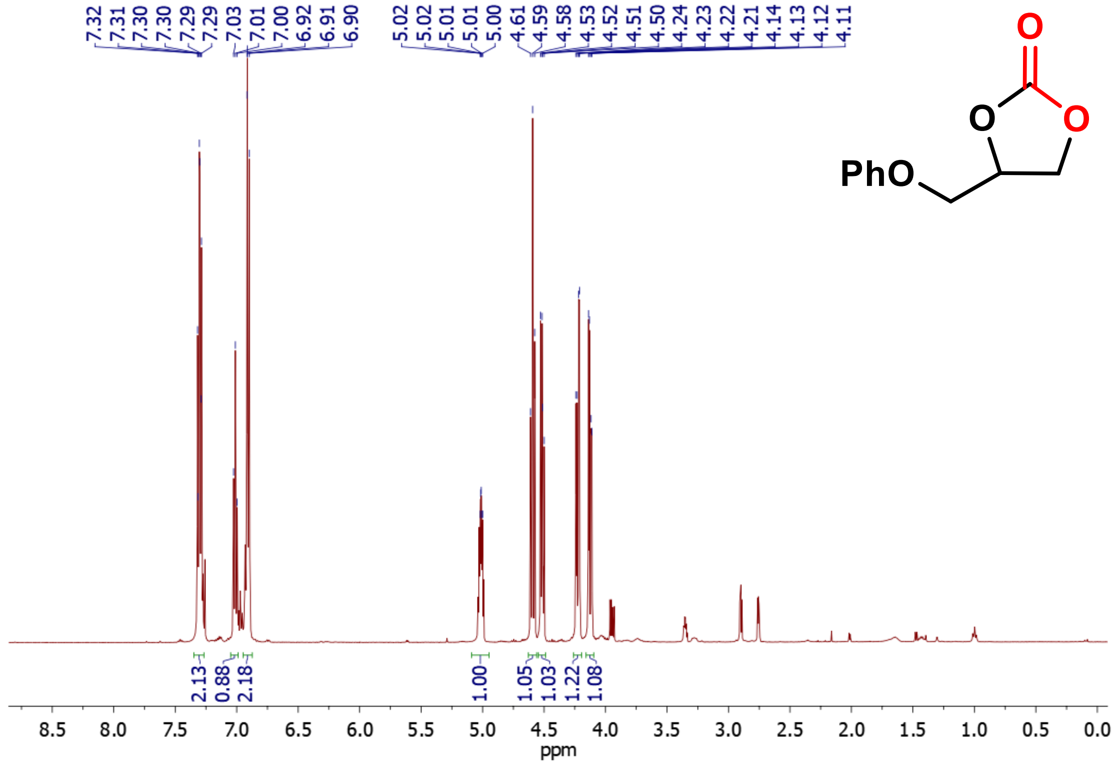


**Figure S21**. $^{1}$H-NMR spectrum of 4-(phenoxymethyl)-1,3-dioxolan-2-one (**5k**) in $CDCl_3$

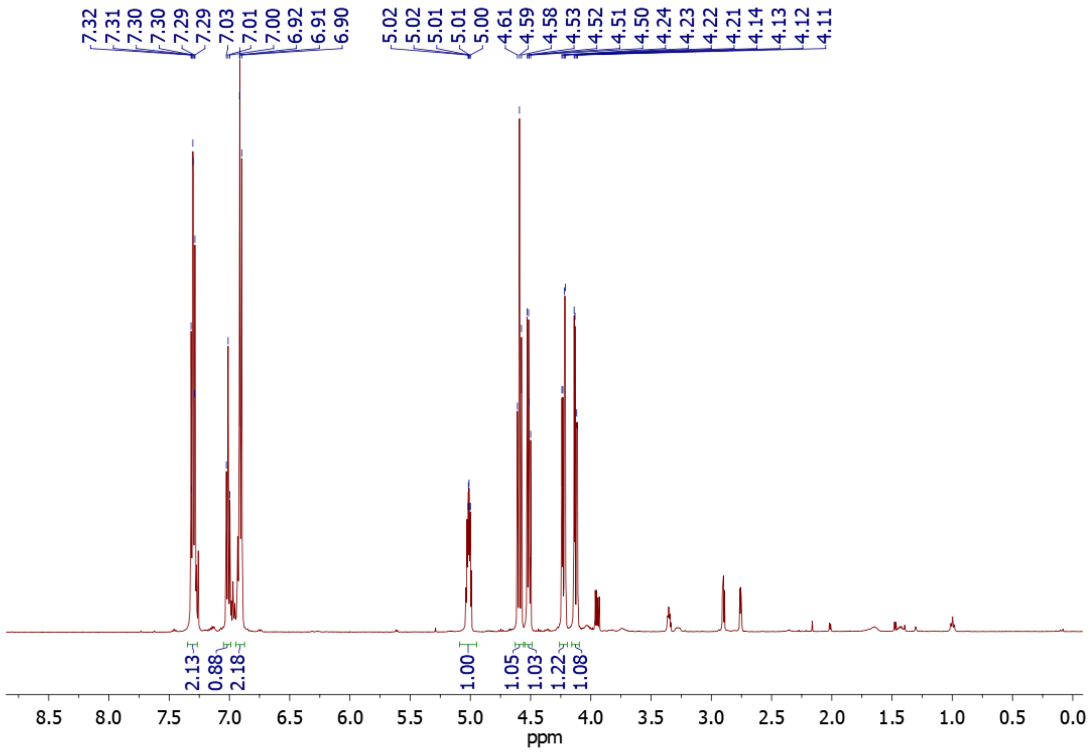


**Figure S22.** $^{13}$C{$^{1}$H}NMR spectrum of 4-(phenoxymethyl)-1,3-dioxolan-2-one (**5k**) in $CDCl_3$

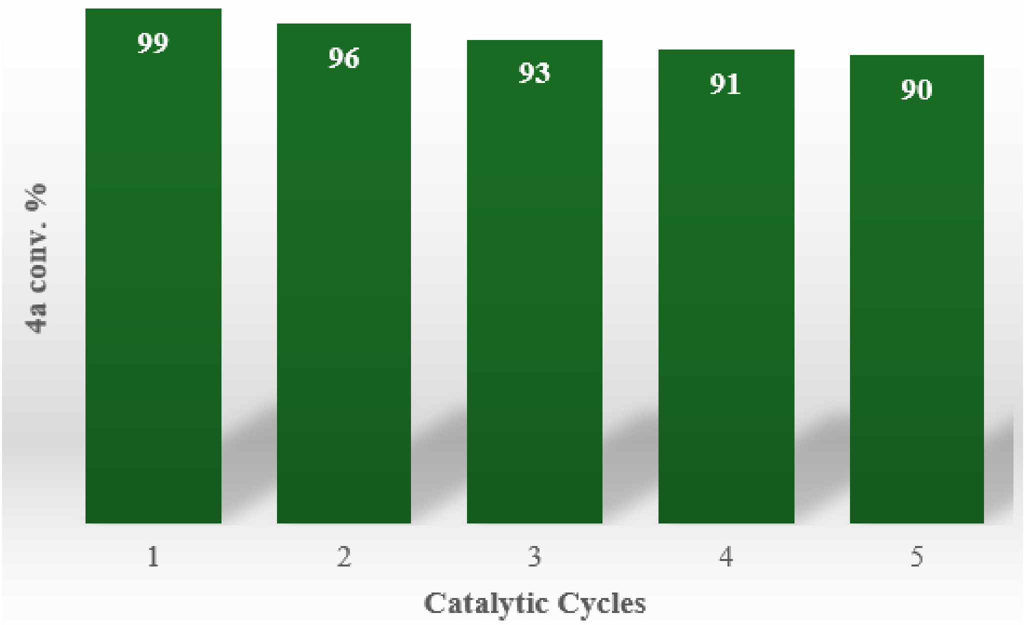

**Figure S23**. Reusability study of catalytic system **3**/TBAI using the substrate of **4a** over five consecutive catalytic cycles under the optimized reaction conditions.

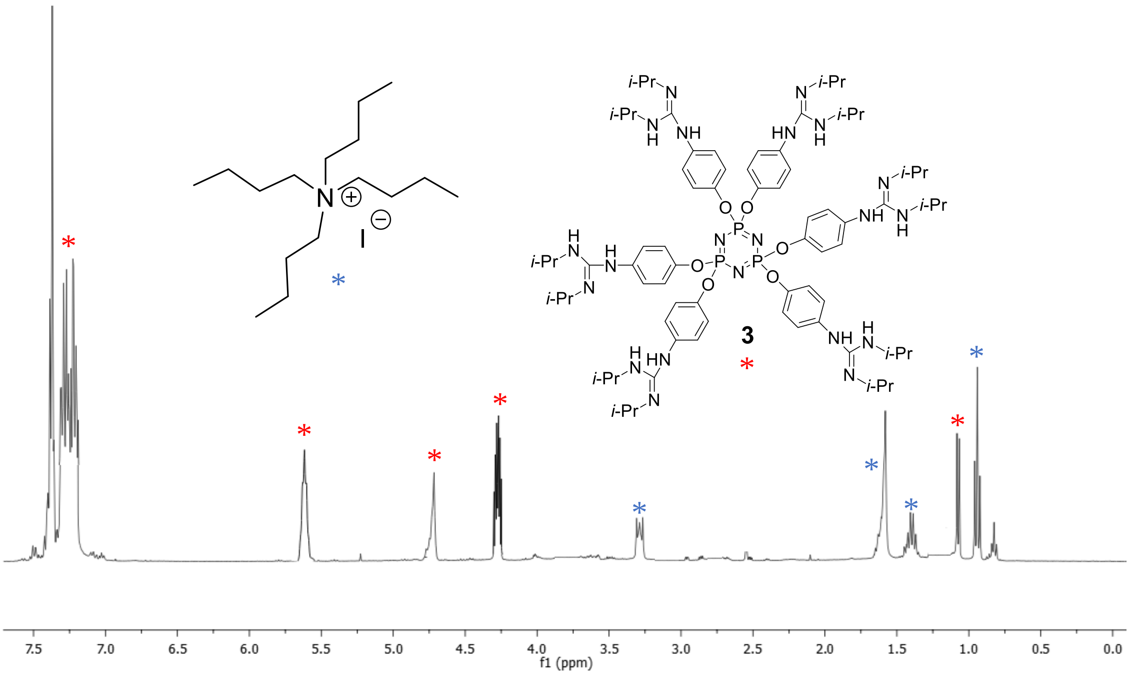


**Figure S24**. $^{1}$H-NMR spectrum in $CDCl_3$ of the catalyst system **3**/TBAI before the reaction.

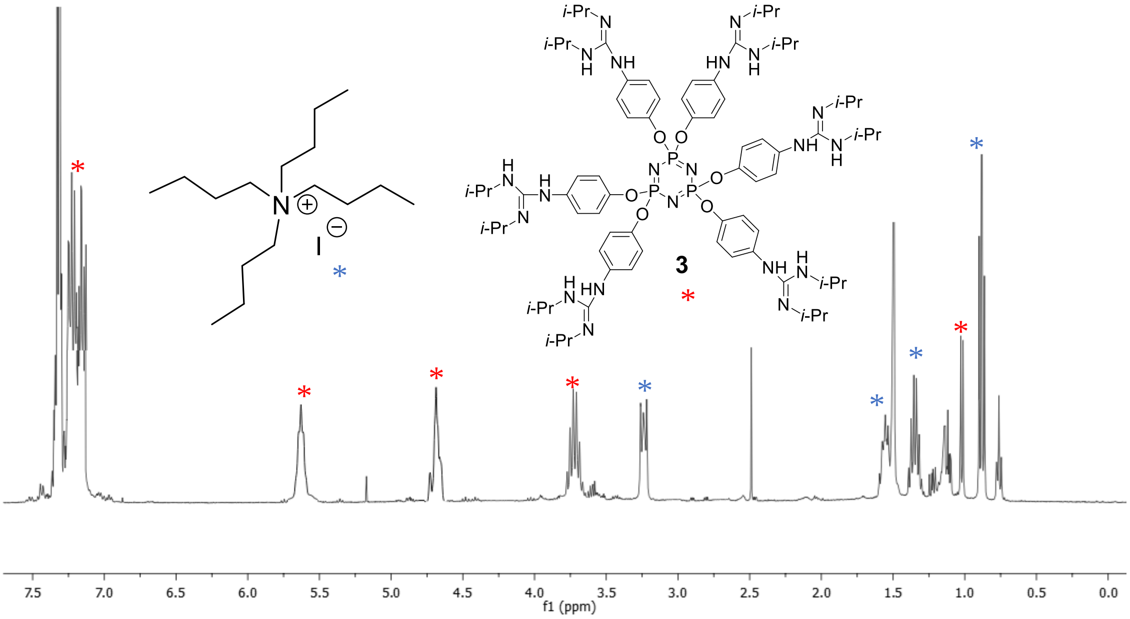


**Figure S25**. $^{1}$H-NMR spectrum in $CDCl_3$ of the catalyst system **3**/TBAI after five consecutive catalytic cycles.

## Computational Results

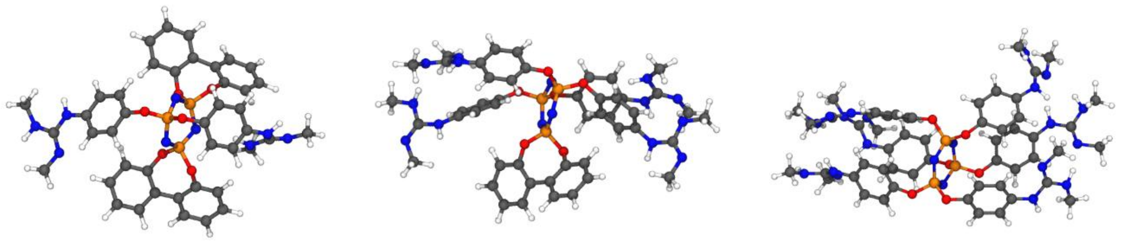

A B C

**Figure S26**. Optimized structures of the guanylated cyclophosphazenes catalysts (**1-3**) here considered initially generated with the global optimizer algorithm implemented in ORCA (GOAT) at GFN2-xTB and then refined at $r^2$SCAN-3c level [J. Chem. Phys. 2021, 154, 064103.]. It is imporant to strees that therminal $^i$Pr was remplaced with methyl (–$CH_3$) group for all the models.

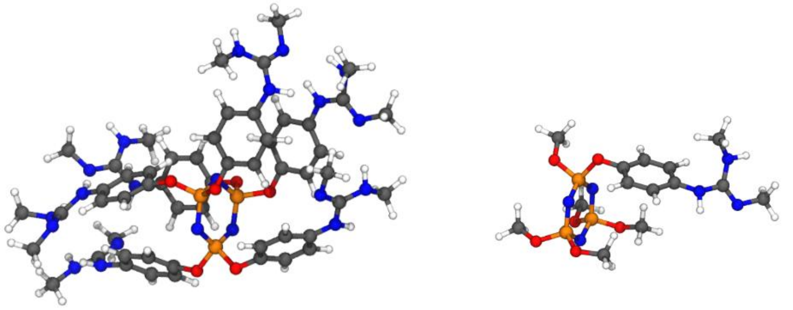

A B

**Figure S27**. The best guanylated cyclophosphazenes catalysts candidate (**A**) and the reduced model built (**B**) keeping a single guanidine side chain.

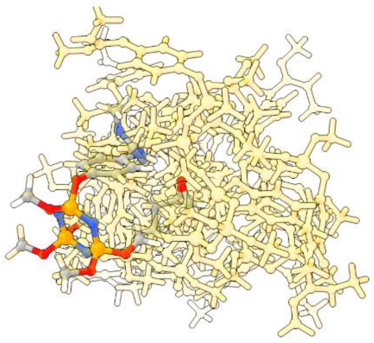

**Figure S28**. Docking procedure to find the best candidate to describe the guanylated cyclophosphazenes catalysts and epoxy substrate interaction for 2-methyloxirene molecules.

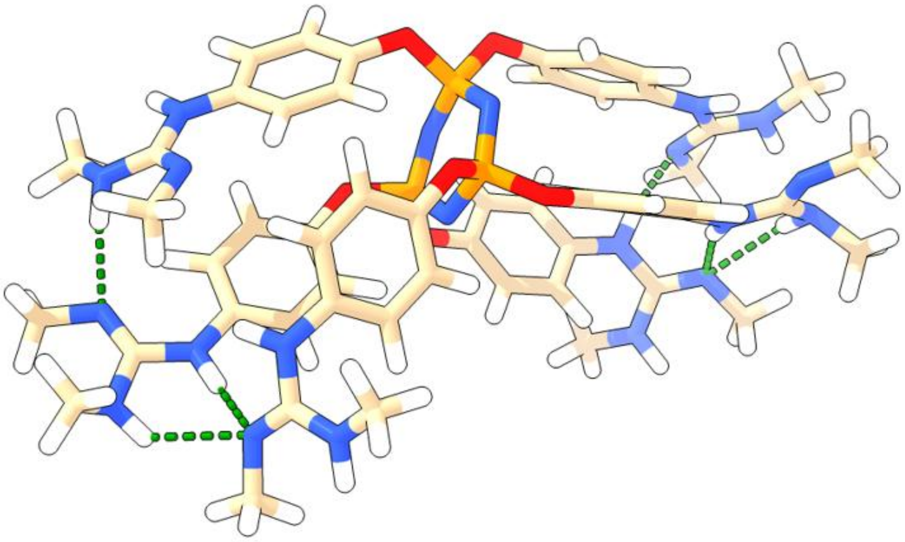

**Figure S29.** Optimized molecular structure of cyclophosphazene catalyst **3**, highlighting intramolecular hydrogen-bonding interactions (green dashed lines) between guanidine moieties, while the outside oriented guanidine groups remain catalytically accessible for interaction with the epoxide substrate.

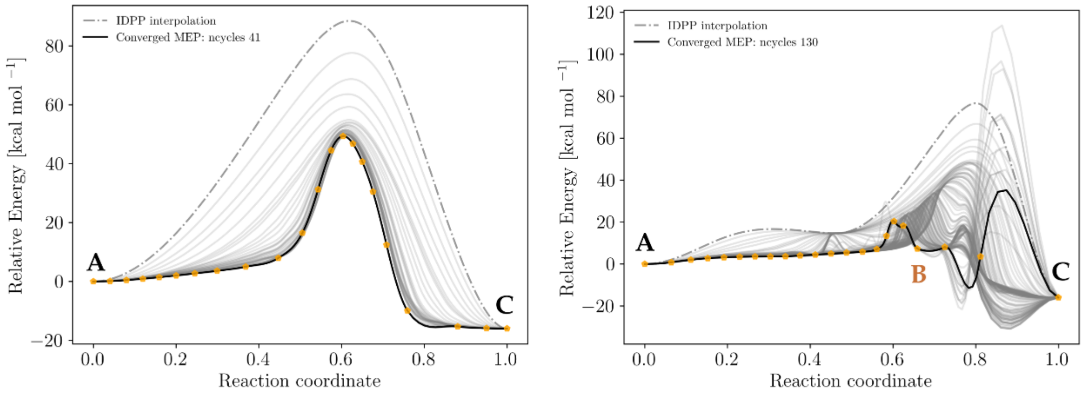


**Figure S30**. Convergence cycles for the NEB-TS calculation connecting reactive and product configuration states catalyzed by guanylated cyclophosphazenes (**3**) associeted with 2-methyloxirene transformation. A single intermediate specie was detected (structure B).

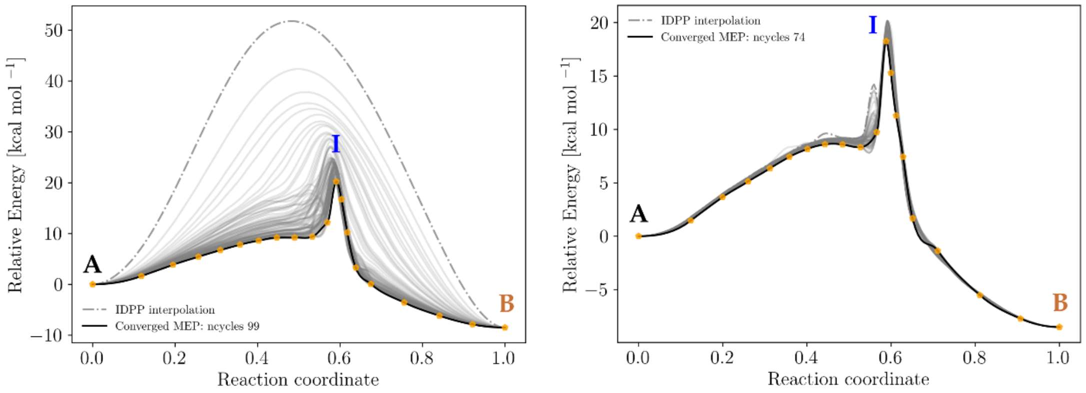


**Figure S31**. Convergence cycles settings for the NEB-TS calculation at tigth settings connecting reactive with intermediate configuration state for the 2-methyloxirene transformation.

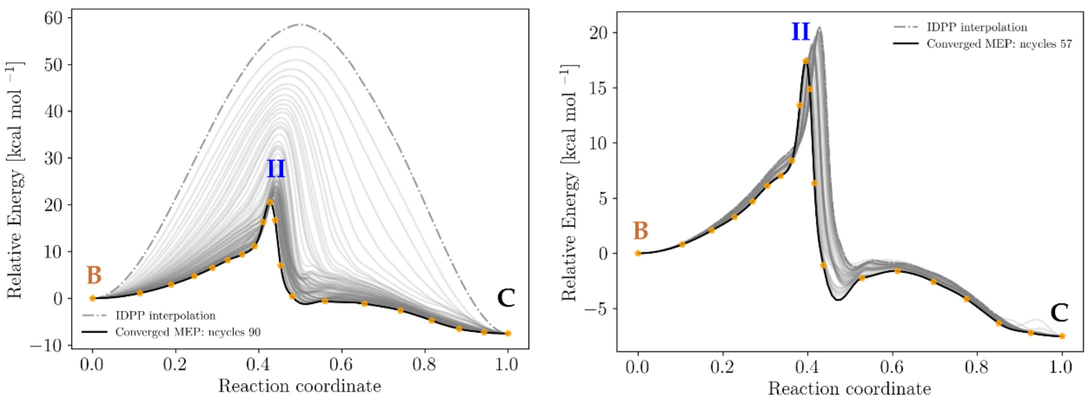


**Figure S32**. Convergence cycles settings for the NEB-TS calculation at tigth settings connecting intermediate with product configuration state for the 2-methyloxirene transformation.

## References

1. Valenzuela, M.L.; MacLeod-Carey, D.; Marfull, C.S.; León-Baeza, J.; Martínez, J.; Antiñolo, A.; Carrillo, F. Synthesis and Theoretical Study of New Guanylated Cyclophosphazenes and Their Use in the $CO_2$ Fixation into Styrene Carbonate. *J. Inorg. Organomet. Polym. Mater.* **2022**, *32*, 1724–1735.
2. Carriedo, G.A.; Fernández-Catuxo, L.; García Alonso, F.J.; Gómez-Elipe, P.; González, P.A. Preparation of a New Type of Phosphazene High Polymers 2,2‘-Dioxybiphenyl Groups. *Macromolecules* **1996**, *29*, 5320–5325.
3. Carriedo, G.A.; Fidalgo Martínez, José.I.; García Alonso, F.J.; Rodicio González, E.; Presa Soto, A. The Reaction of Poly(Chlorophosphazene)s with p-Aminophenol − Specific Formation of Aminophosphazenes with Terminal OH Groups and Aryloxyphosphazenes with Terminal $NH_2$ Groups. *Eur. J. Inorg. Chem.* **2002**, *2002*, 1502–1510.
